# Folding and fluorescence enhancement with strong odd–even effect for a series of merocyanine dye oligomers†

**DOI:** 10.1039/d1sc01678d

**Published:** 2021-05-20

**Authors:** Xiaobo Hu, Alexander Schulz, Joachim O. Lindner, Matthias Grüne, David Bialas, Frank Würthner

**Affiliations:** Institut für Organische Chemie, Universität Würzburg Am Hubland 97074 Würzburg Germany; Center for Nanosystems Chemistry (CNC), Universität Würzburg Theodor-Boveri-Weg 97074 Würzburg Germany wuerthner@uni-wuerzburg.de

## Abstract

A series of merocyanine (MC) oligomers with a varying number of chromophores from two to six has been synthesized *via* a peptide synthesis strategy. Solvent-dependent UV/vis spectroscopic studies reveal folding processes for the MC oligomers driven by strong dipole–dipole interactions resulting in well-defined π-stacks with antiparallel orientation of the dyes. Whilst even-numbered tetramer **4** and hexamer **6** only show partial folding into dimeric units, odd-numbered trimer **3** and pentamer **5** fold into π-stacks of three and five MC units upon decreasing solvent polarity. In-depth 2D NMR studies provided insight into the supramolecular structure. For trimer **3**, an NMR structure could be generated revealing the presence of a well-defined triple π-stack in the folded state. Concomitant with folding, the fluorescence quantum yield is increased for all MC oligomers in comparison to the single chromophore. Based on radiative and non-radiative decay rates, this fluorescence enhancement can be attributed to the rigidification of the chromophores within the π-stacks that affords a pronounced decrease of the non-radiative decay rates. Theoretical investigations for the double and triple dye stacks based on time-dependent density functional theory (TD-DFT) calculations indicate for trimer **3** a pronounced mixing of Frenkel and charge transfer (CT) states. This leads to significant deviations from the predictions obtained by the molecular exciton theory which only accounts for the Coulomb interaction between the transition dipole moments of the chromophores.

## Abstract 2

A series of merocyanine (MC) oligomers with a varying number of chromophores from two to six has been synthesized *via* a peptide synthesis strategy.

## Graphical abstract

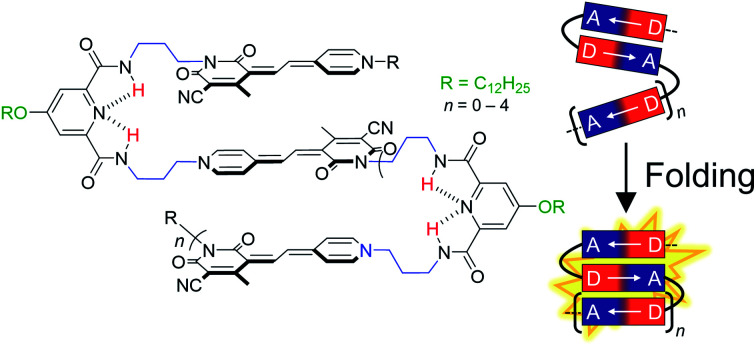

## Introduction

Dye aggregates currently receive increasing attention due to their high relevance for a variety of applications, *e.g.* in organic photovoltaics,^1,2^ organic light-emitting devices^3,4^ and light-harvesting systems for artificial photosynthesis.^5,6^ In order to gain fundamental understanding of the intermolecular interactions leading to the desired functional properties, well-defined aggregates with precise control of the chromophore arrangement and aggregate size are desired.^7–9^ The importance of defined chromophore arrangements is also demonstrated by natural light-harvesting complexes, in which photosynthetic pigments are organized into discrete structures stabilized by a protein shell, which ensures an efficient light-harvesting process and charge separation.^10,11^ However, it is obvious that the realization of such complex structures is beyond the scope of current supramolecular chemistry. Therefore, different strategies have been developed for the investigation of dye–dye interactions and their impact on the functional properties. For example, the covalent linkage of chromophores within cyclophanes^12–15^ and macrocycles^16,17^ by appropriate linker moieties guarantees a distinct orientation and distance, which allows in-depth studies on the optical and electronic properties. Unfortunately, the realization of π-stacks with more than two chromophores is not feasible with this approach. In this regard, DNA-templated self-assembly has been successfully employed, leading to the formation of defined dye stacks of different size.^18–20^ Nonetheless, these studies are in general restricted to aqueous solvents due to the negatively charged DNA backbone, which may complicate spectroscopic studies due to an active participation of water by *e.g.* proton transfer processes. Hence, a promising strategy to avoid the above-mentioned drawbacks represents the foldamer approach,^21^ where a specific number of chromophores are covalently linked through appropriate spacer units.^22–25^ This approach has found widespread adoption and has been successfully employed in the formation of donor–acceptor stacks,^26,27^ artificial β-sheet structures^28^ and catalytically active chromophore stacks.^18^ In contrast to the aforementioned approaches, the folding of the desired dye aggregates can be controlled by external stimuli, *e.g.* the addition of ions^29^ or change of the solvent polarity.^30^ In this way, well-defined dye aggregates with specific orientations of the chromophores can be realized.

**Fig. 1:**
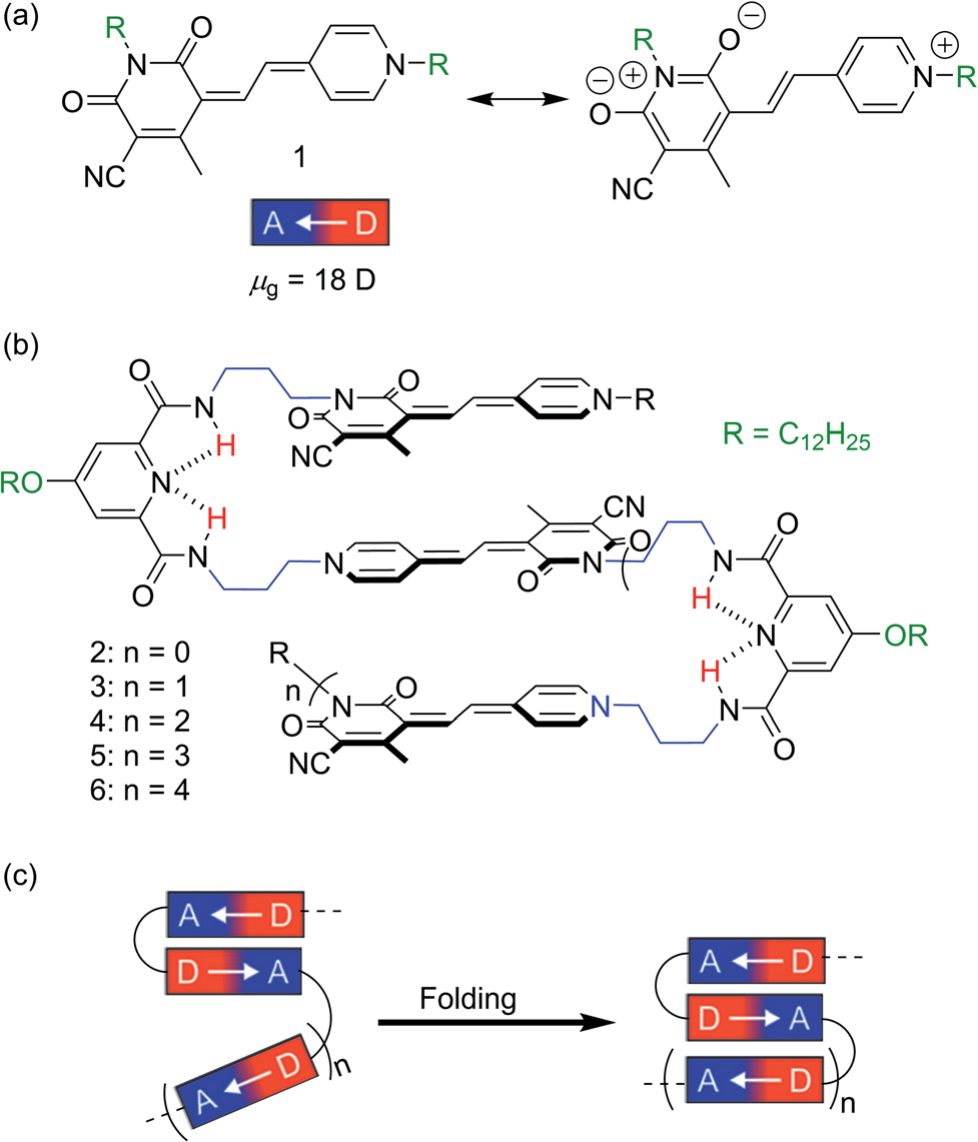
(a) Resonance structures and donor–acceptor character with large dipole moment of reference merocyanine **1**. (b) Chemical structure of MC oligomers **2–6** in the folded state. The dashed lines indicate the hydrogen bonding between the amide protons and the pyridine nitrogen atom of the linker moiety. (c) Schematic representation of the folding process of **2–6** into discrete π-stacks driven by dipole–dipole interactions.

Recently, we have reported on the stepwise hierarchical folding of merocyanine (MC) pentamer **5** (Fig. 1b) from partially folded monomeric and dimeric MC subunits to a completely folded structure upon decreasing solvent polarity.^31^ The utilized MC chromophore exhibits a significant zwitterionic character (Fig. 1a) resulting in a large ground state dipole moment (*μ*_g_ = 18 D), which gives rise to strong interchromophoric dipole–dipole interactions.^32^ Consequently, well-defined quintuple π-stacks with antiparallel orientations of the chromophores were obtained for pentamer **5** in non-polar solvents.^31^

In order to understand the prerequisites for such a hierarchical structure formation process of MC based foldamers, we have now synthesized a series of MC oligomers **2–4** and **6**. Thus, together with the previously reported pentamer **5**, we have a consecutive sequence of MC foldamers with various size (dimer to hexamer, Fig. 1b) and can elucidate structure–property relationships in non-polar solvents by UV/vis, fluorescence and NMR spectroscopy. Notably, whereas trimer **3** and pentamer **5** form triple and quintuple stacks in non-polar solvent, respectively, tetramer **4** and hexamer **6** containing even numbers of MC units undergo only partial folding resulting in the formation of MC dimer subunits as present for dimer **2**.

## Results and discussion

### Molecular design and synthesis

**Scheme 1:**
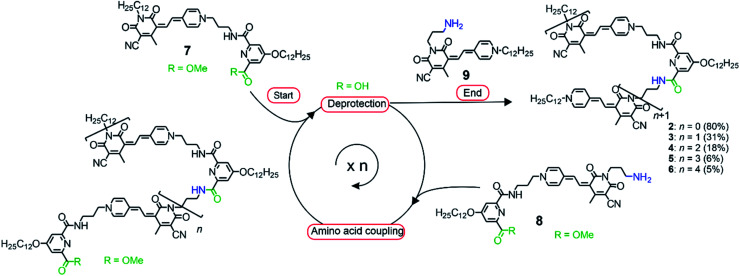
Synthetic route towards MC oligomers **2–6** employing a peptide synthesis strategy starting with the deprotection of the carboxylic group of precursor **7** and subsequent amino acid coupling with compound **8**. The procedure was repeated until the desired oligomer length was obtained. Finally, the target compound was obtained by condensation reaction with building block **9** in the last step. Reaction conditions for the deprotection step: NaOH_aq_, THF/MeOH, 2 h; reaction conditions for the amino acid coupling: HBTU, DIPEA, CH_2_Cl_2_, 1 h.

In order to direct a stepwise folding into well-defined merocyanine stacks, a sophisticated design of the molecular backbone is required. Hence, our employed spacer moieties were selected based on the following considerations: (1) the diamide pyridine motif^33,34^ connecting the MC units should support the dipolar aggregation of the chromophores *via* a hydrogen-bonded turn motif (Fig. 1b). (2) The length of the short alkyl spacer containing three carbon atoms (–(CH_2_)_3_–, highlighted in blue) was chosen to avoid ill-folding (for shorter alkyl chains) or too much structural flexibility (for longer alkyl chains). (3) The solubilizing side chains (–OC_12_H_25_ highlighted in green) at the pyridine moiety provide solubility in less polar solvents, in which the dipole–dipole interaction is maximized. (4) The diamide structure enables the use of efficient and reliable amino acid coupling reactions as done in peptide synthesis and thus, should facilitate oligomer prolongation. Accordingly, MC oligomers **2–6** were synthesized by a peptide synthesis strategy from the previously reported building blocks **7–9** (Scheme 1), which have been used for the synthesis of pentamer **5** in our earlier work.^31^ As a first step, the carboxylic ester moiety of **7** was deprotected with aqueous NaOH and the resulting acid reacted with precursor **8**, which contains both an amine and a protected acid group. This cycle of deprotection and coupling was repeated until the desired oligomer length was obtained. In the last amino acid coupling step, compound **9** was used instead of **8** to afford the target compound carrying a dodecyl chain on the terminal donor and the acceptor group. All target compounds were purified by column chromatography, followed by gel permeation chromatography to give the MC oligomers with a decreasing overall yield from 80% for dimer **2** to 5% for hexamer **6** based on the starting monomer **7**. The synthesis of reference dye **1** has been previously reported.^35^ For details on the synthetic procedure and characterization of the new compounds we refer to the ESI.†

### Solvent-dependent folding revealed by UV/vis absorption spectroscopy

**Fig. 2:**
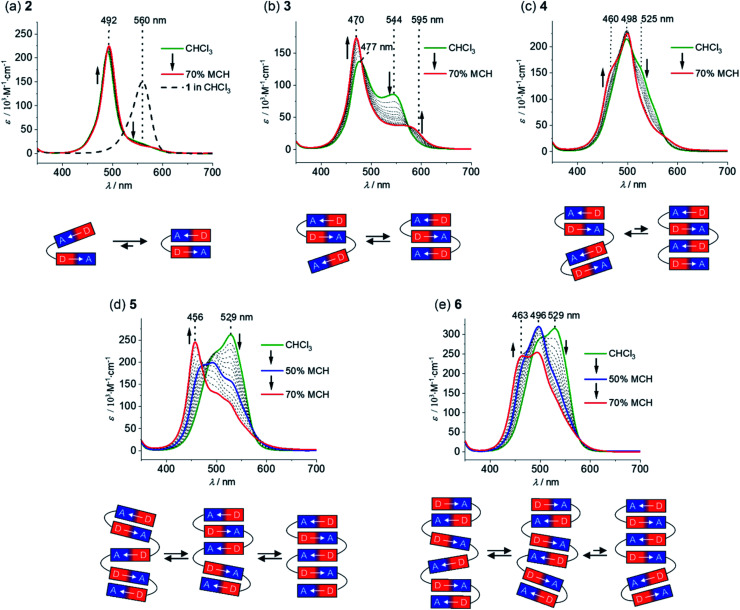
Solvent-dependent UV/vis spectra of MC oligomers (a) **2**, (b) **3**, (c) **4**, (d) **5** and (e) **6** in CHCl_3_/MCH solvent mixtures at 20 °C (*c* ∼ 10^−6^ M) starting from pure CHCl_3_ (green line) with stepwise increase of the amount of methylcyclohexane to 70% (red line). The arrows indicate the spectral changes upon decreasing solvent polarity and the dashed spectrum in panel (a) shows the absorption spectrum of reference chromophore **1** in pure CHCl_3_. In addition, the schematic representation of the folding process is displayed below the respective UV/vis spectra.

The folding process of MC oligomers **2–6** was investigated by UV/vis absorption and steady-state fluorescence spectroscopy. In general, the aggregation tendency of merocyanine dyes is significantly influenced by the solvent polarity,^32^*i.e.* a polar solvent interacts favorably with the dipolar dyes and therefore leads to a lower driving force for folding, whereas the formation of folded structures (or self-assembled aggregates) is favored in non-polar solvents due to the increased strength of the electrostatic dipole–dipole interactions. Therefore, we have performed solvent-dependent UV/vis studies in order to investigate the folding process of **2–6**. The absorption spectrum of dimer **2** in pure chloroform, a solvent of intermediate polarity (*ε*_r_ ∼ 4.81),^36^ shows an intense and sharp absorption band at 492 nm (Fig. 2a, green line), which is hypsochromically shifted in comparison to the monomer absorption band (black dashed line). This is highly indicative for the presence of a tightly π-stacked dimeric motif^35^ with the hypsochromic shift arising from H-type exciton coupling between the transition dipole moments of the MC chromophores.^37^ Since no spectral changes are observed upon variation of the concentration (Fig. S13a†), we can exclude intermolecular self-assembly and therefore assign the hypsochromically shifted absorption band to a folded structure with a stack of two merocyanine chromophores MC_2_ (Fig. 2a, bottom). Notably, the absorption spectrum of dimer **2** indicates the presence of mainly folded species, since no pronounced absorption can be detected in the region of the monomer absorption band at ∼560 nm. A decrease of the solvent polarity upon the addition of methylcyclohexane (MCH, *ε*_r_ ∼ 2.07)^38^ does not affect the absorption spectrum (Fig. 2a, red line), revealing that complete folding is already accomplished in chloroform. Hence, the strong folding tendency of **2** indeed proves our proper choice of the molecular turn for the formation of well-defined π-stacks in solution.

The spectrum of trimer **3** in chloroform is characterized by a further blue-shifted absorption band at 477 nm (Fig. 2b, green line), indicating the presence of merocyanine stacks larger than a dimer, *i.e.* a triple stack. However, one can notice an additional, pronounced absorption band at 544 nm, which is slightly blue-shifted with respect to the monomer absorption band. We attribute this to the co-existence of a partially folded structure MC_2_–MC comprising one double stack and a loosely bound “monomeric” chromophore with weak exciton coupling causing the slightly blue-shifted absorption at 544 nm (Fig. 2b). Upon decreasing the solvent polarity, the intensity of the absorption band at longer wavelengths decreases with a concomitant increase and narrowing of the absorption band at shorter wavelengths (Fig. 2b). Both of these features indicate an increasing degree of order of the triple stack in the lower polarity environment. Accordingly, the equilibrium has shifted towards the fully folded species upon increasing the amount of MCH. The absorption spectrum in CHCl_3_/MCH 30 : 70 displays an intense absorption band at 470 nm and a significantly weaker band at 595 nm, which is bathochromically shifted compared to the monomer absorption (Fig. 2b, red line) and may arise from a partially allowed excitation of lower J-type exciton states.^39^ Remarkably, in the case of tetramer **4**, the main absorption band is not further hypsochromically shifted as one would expect in the case of a quadruple dye stack (Fig. 2c, green line). Instead, the main absorption band in chloroform is located at 498 nm, indicating the presence of pairwise merocyanine stacks (MC_2_–MC_2_). The absence of quadruple dye stacks, whose absorption maximum should be located at ∼460 nm,^40^ can be rationalized by the weak electrostatic attraction between the dimeric units that are not attracted anymore by dipole–dipole interactions (Fig. 2c, bottom). Even upon increasing the amount of MCH up to 70%, only minor changes are observed in the absorption spectrum of **4** (Fig. 2c, red line). Thus, the absorption at ∼525 nm is decreasing accompanied by a subtle increase at ∼460 nm, which indicates only a partial folding into quadruple stacks.

The spectral changes observed for pentamer **5** reveal an interesting stepwise folding into a quintuple dye stack (Fig. 2d).^31^ The spectrum in pure chloroform exhibits two absorption bands at 490 and 529 nm (Fig. 2d, green line), that can be rationalized by a folded state comprising double stack(s) and single chromophore(s) with weak H-type coupling (Fig. 2d, bottom), resembling the situation observed for trimer **3**. Decrease of the solvent polarity upon increasing the amount of MCH results in a loss of intensity for the low-energy absorption band for pentamer **5** accompanied by a further hypsochromic shift of the high-energy absorption band. Hence, the spectrum in chloroform/MCH 50 : 50 shows an additional absorption band at 470 nm (Fig. 2d, blue line), giving hint for a folding into a triple stack (Fig. 2d, bottom) as in the case of trimer **3**. Upon further decreasing the solvent polarity (CHCl_3_/MCH 30 : 70, Fig. 2d, red line), a continuing hypsochromic shift of the main absorption band can be observed (456 nm), that can be assigned to a folded structure containing a stack of five chromophores (Fig. 2d, bottom).^31^

The spectrum of hexamer **6** in pure chloroform closely resembles the one of pentamer **5** indicating the presence of double stacks and single chromophores (Fig. 2e, green line). Upon decreasing the solvent polarity to CHCl_3_/MCH 50 : 50 (Fig. 2e, blue line), the absorption band at 529 nm decreases and the band at 496 nm gains intensity. As in the case of tetramer **4**, this can be rationalized by the presence of multiple MC_2_ units. Further decreasing the solvent polarity (CHCl_3_/MCH 30 : 70, Fig. 2e, red line) leads to the emergence of a further hypsochromically shifted shoulder at 463 nm, indicating the formation of a folded state comprising a quadruple and a dimeric stack of merocyanine dyes.

### Structural elucidation of the folded states by NMR spectroscopy

**Fig. 3:**
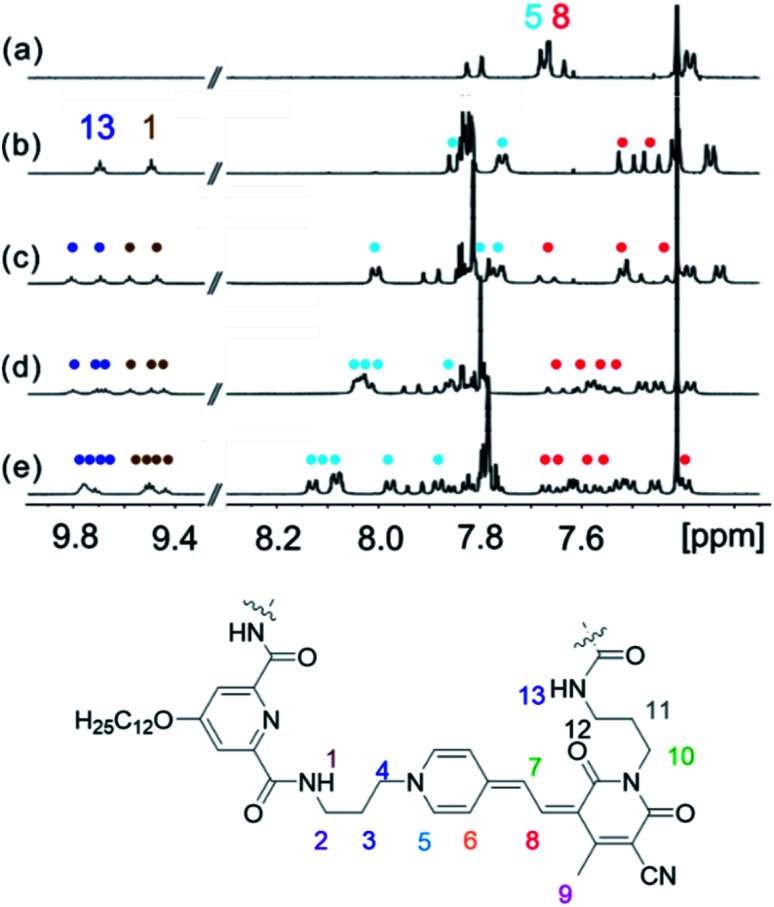
Excerpts of the ^1^H NMR spectra (400 MHz, amide and aromatic region) of (a) monomer **1**, (b) dimer **2**, (c) trimer **3**, (d) tetramer **4** and (e) pentamer **5** in CDCl_3_ at 295 K.

NMR spectroscopy is a powerful tool for the investigation of supramolecular structures in solution.^41,42,44^ Thus, we have performed ^1^H NMR studies for MC oligomers **2–5** to gain deeper insight into the chromophore arrangement in the folded state (hexamer **6** showed a significant broadening of the ^1^H NMR signals, so that an unambiguous assignment to the protons was not possible). Due to the low solubility of the compounds in methylcyclohexane, the measurements were performed in pure CDCl_3_ to reach the required concentration (∼10^−3^ M). The ^1^H NMR spectra of oligomers **2–5** show a significant down- or upfield shift of the aromatic protons of the merocyanine chromophores with respect to the single chromophore (Fig. 3), which indeed indicates the presence of π-stacked merocyanine chromophores and results from (de)shielding due to the ring current of the adjacent chromophores.^43,44^ Notably, the signals of the amide protons 1 and 13 (9.4–9.8 ppm) undergo a significant downfield shift in comparison to the spectrum of the diamide pyridine moiety without MC units (∼7.7 ppm),^31^ which can be attributed to the constrained conformation and thus strengthening of the hydrogen bonding due to folding as displayed in Fig. 1b. Remarkably, the signals in the aromatic region are well-resolved (Fig. 3b–e), being indicative of the presence of defined species.

**Fig. 4:**
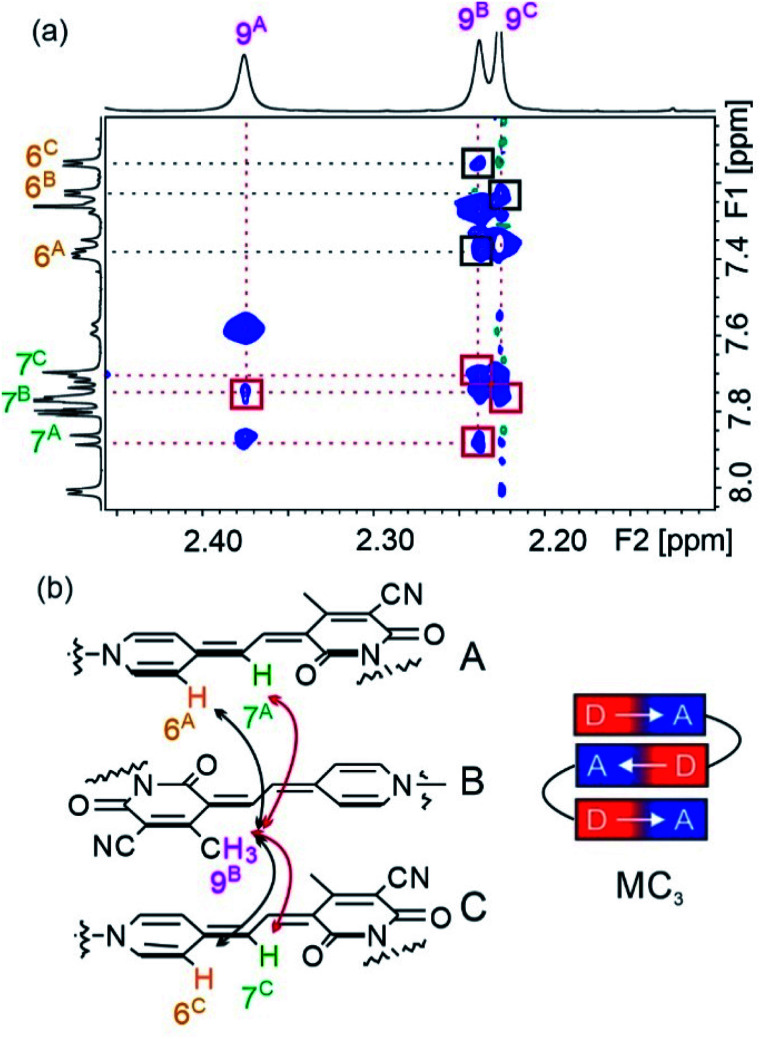
(a) Excerpt of the ^1^H–^1^H ROESY spectrum (600 MHz) of trimer **3** in CDCl_3_ at 295 K. Positive and negative signals are displayed in blue and green, respectively. (b) Schematic representation of the triple stack of **3** with antiparallel orientation of the chromophores. The correlations between protons 7 ↔ 9 (red rectangles in the ^1^H–^1^H ROESY spectrum) of neighboring MCs are indicated by red double-headed arrows and 6 ↔ 9 correlations (black rectangles in the ^1^H–^1^H ROESY spectrum) by black double-headed arrows. Only one set of correlation is shown for clarity.

In order to prove the presence of π-stacks in chloroform, ^1^H–^1^H rotating-frame Overhauser effect spectroscopy (ROESY) and ^1^H–^1^H nuclear Overhauser effect spectroscopy (NOESY) were performed, which enable us to identify protons in close spatial proximity. Indeed, cross-peaks between methyl protons 9 and methine protons 7 of different chromophores can be observed for oligomers **2–5** (Fig. 4 and S7†), confirming a π-stacked arrangement of the merocyanine chromophores with antiparallel orientation. Accordingly, the presence of a double (Fig. S7†) and triple stack (Fig. 4) can be proven for oligomers **2** and **3** in chloroform, respectively. Notably, in the case of tetramer **4**, only two pairs of cross-signals are obtained, that can be rationalized by the presence of two pairwise stacks in chloroform rather than a quadruple stack (Fig. S8†), in agreement with the results obtained from UV/vis spectroscopy (*vide supra*). In a similar manner, correlations between three different signals for proton 9 and proton 6 are observed in the NOESY spectrum of pentamer **5**, indicating a partially folded structure in chloroform with two MC_2_ subunits and one loosely stacked chromophore in the center (Fig. S9†).^31^

**Fig. 5:**
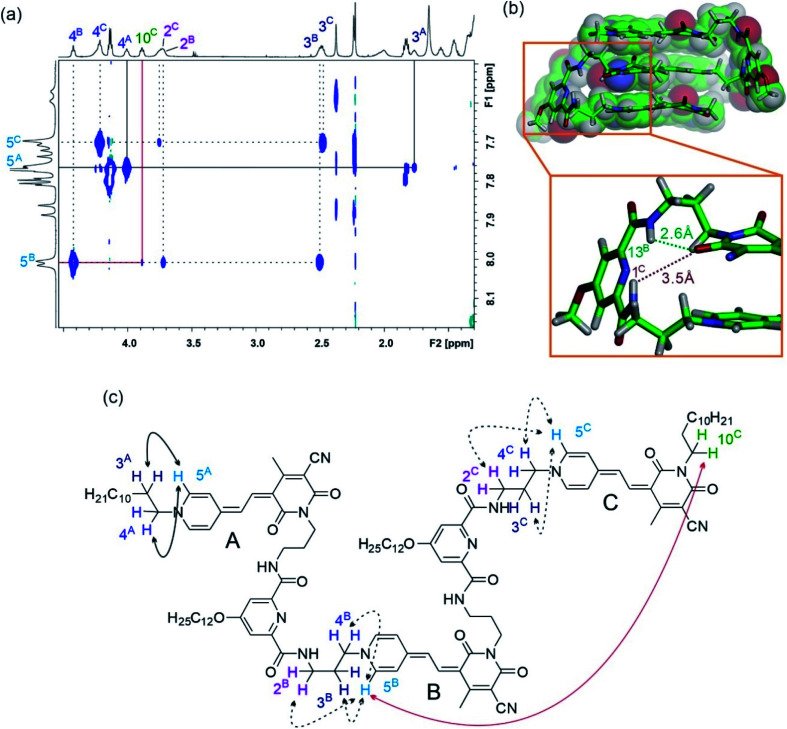
(a) Excerpt of the ^1^H–^1^H ROESY spectrum (600 MHz) of trimer **3** in CDCl_3_ at 295 K. (b) Simulated NMR structure of **3** based on ^1^H–^1^H ROESY data (carbon: green, oxygen; red, nitrogen: blue and hydrogen: white) with enlarged section showing the hydrogen bonds between the carbonyl oxygen atom of the merocyanine acceptor moiety and nearby amide protons 1^C^ (brown) and 13^B^ (green). Dodecyl chains are replaced by methyl groups for clarity. (c) Chemical structure of **3** with significant correlations between protons indicated by double-headed arrows.

The well-resolved signals and correlations in the ROESY spectrum of trimer **3** enabled us to generate an NMR structure of the trimeric stack as a representative example. In the following, we briefly describe our procedure for the assignment of the protons exemplified by some chosen correlations. As a first step, ^1^H–^1^H correlation spectroscopy (COSY) was performed in order to classify protons within the same spin system (Fig. S2†). As a next step, correlations in the ^1^H–^1^H ROESY spectrum allowed us to assign the signal sets to the corresponding merocyanine chromophore. Accordingly, we could identify the set of signals arising from chromophore B in the interior of the stack, since it displays correlations with the protons of the two chromophores A and C in the exterior (7^A^ ↔ 9^B^ ↔ 7^C^, see Fig. 4), whereas A and C each display only correlations to one chromophore, *i.e.* chromophore B. Moreover, the 3^A^ ↔ 5^A^ correlation revealed the signals of the protons pertaining to the pyridine unit located on chromophore A (Fig. 5a and c, black line). In this way, the two sets of signals corresponding to chromophores A and B have been identified and the remaining set could be assigned to chromophore C. Finally, the signals of the linker protons were allocated based on the cross-peaks in the ROESY spectrum with the protons of the adjacent merocyanine units and the correlations within the spacer (Fig. 5 and S4†).

The resulting NMR structure (for details see the ESI†) shows the expected triple π-stack of the merocyanine chromophores with antiparallel orientation and an intermolecular distance of ∼3.5 Å (Fig. 5b). Notably, the structure suggests hydrogen bonds between the amide protons of the spacer unit and the adjacent carbonyl oxygen atom of the merocyanine moiety of different lengths of 2.6 Å (green dotted line) and 3.5 Å (brown dotted line). This is in agreement with the distinct chemical shifts of the amide protons observed in the ^1^H NMR spectrum, revealing a more pronounced downfield shift in the case of protons 13 compared to protons 1 due to the stronger hydrogen bonding (Fig. 3c). The same holds true for the other spacer moiety in trimer **3**. Hence, our in-depth NMR studies are in agreement with the results obtained from UV/vis spectroscopy in chloroform, which indicate a folded arrangement for trimer **3** into triple stacks as well as the presence of double stacks (MC_2_) in the case of MC oligomers **2**, **4** and **5**.

### Electronic coupling in folded π-stacks according to TD-DFT calculations and the molecular exciton model

**Fig. 6:**
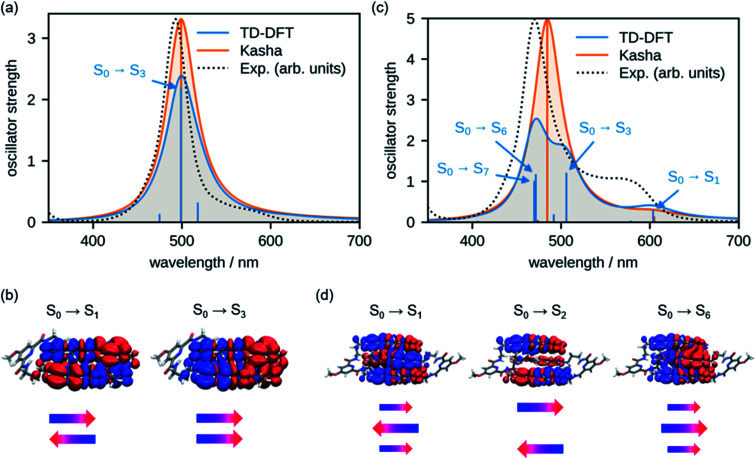
Calculated UV/vis spectra of (a) **2** and (c) **3** on the TD-DFT level of theory^45,46^ and the molecular exciton model of interacting transition dipole moments according to Kasha.^37^ For both methods, vertical transitions are displayed as sticks and convoluted spectra are given as solid lines. Transitions with significant oscillator strength were assigned with arrows. TD-DFT transition densities of the most relevant states are plotted for (b) **2** and (d) **3**. Additionally, schematic representations of orientation and magnitude of monomer transition dipole moments are depicted as obtained by Kasha theory.

In order to rationalize the spectral changes observed upon folding, we have calculated the UV/vis absorption spectra for the double and triple stack of oligomers **2** and **3**, respectively. For this, the geometries were optimized at the level of density functional theory (DFT) employing the def2-SVP^45^ basis set and the B3LYP-D3 functional,^46^ which also includes Grimme's dispersion correction.^47^ The spectra were simulated by means of time-dependent density functional theory (TD-DFT, CAM-B3LYP-D3/def2-SVP) using the geometry-optimized structures. Accordingly, the shape of the experimental spectra (Fig. 6a and b, black dotted lines) are well reproduced by the simulations (blue line). Note that the calculated spectra were shifted by 0.491 eV towards lower energies to compensate the overestimation of the transition energies by the employed functional. For comparison, we have also calculated the absorption spectrum with the results obtained from molecular exciton theory (for details see the ESI†), which only considers Coulomb interaction between the transition dipole moments (orange line). In accordance with experimental results, the simulations of both methods show an intense absorption band for dimer **2**. The transition density for the S_0_ → S_1_ and S_0_ → S_3_ obtained from TD-DFT calculations can be regarded as a forbidden out-of-phase and as an allowed in-phase coupling of the transition densities of the individual chromophores, respectively (Fig. 6b). This is in agreement with molecular exciton theory that predicts an H-type coupling between the chromophores in cofacial π-stacks^30^ because only the transition to the higher excited state is allowed. Notably, in contrast to the molecular exciton theory, the results from TD-DFT calculations reveal the existence of additional S_0_ → S_2_ and S_0_ → S_4_ transitions (Fig. S19†) with a dominant charge-transfer (CT) character, however, with negligible oscillator strength (Table S4†).

As shown in our UV/vis absorption experiments for trimer **3**, a further hypsochromic shift of the absorption is observed compared to dimer **2** in the folded state. According to our TD-DFT calculations, the low energy transitions S_0_ → S_1_ and S_0_ → S_2_ as well as transition S_0_ → S_6_ can be interpreted as a Coulomb coupling of the transition dipole moments of the individual chromophores with no significant CT character (Fig. 6d). Hence, the transition to the lowest excited state exhibits only a small oscillator strength due to the out-of-phase coupling of the transition dipole moments. However, it is important to emphasize that the total transition dipole moment is not fully cancelled for this lowest energy state due to the higher coefficient for the chromophore in the interior of the triple stack as indicated by the larger arrow in Fig. 6d. Accordingly, differently from the dimer, a non-vanishing oscillator strength prevails for the lowest energy excitonic state. The next transition to the second excited state is fully forbidden since the transition dipole moments of the two outer chromophores show an out-of-phase coupling whilst the transition S_0_ → S_6_ transition has a large oscillator strength arising from the in-phase coupling of the transition dipole moments, which gives rise to the intense absorption band at shorter wavelengths. These results are in accordance with Kasha's molecular exciton theory. However, our results obtained from TD-DFT calculations suggest the existence of several further transitions with pronounced CT character (Fig. 6c and Table S5†) with S_0_ → S_3_ and S_0_ → S_7_ bearing significant oscillator strength, thereby indicating a strong mixing between the Frenkel and CT states.

The comparison of the calculated and experimental spectrum of **2** (Fig. 6a, black dotted line) shows that both molecular exciton theory and TD-DFT calculations are able to reproduce the absorption spectrum. In the case of trimer **3**, however, we can observe differences between the experimental data and the spectra calculated by TD-DFT. The results obtained by molecular exciton theory show a better match to the experiment with deviations mainly in the intensity and shape of the lower energy band, presumably due to the neglect of vibronic coupling.^48^ In the case of TD-DFT, the main absorption peak of the trimer is split in two bands due to the mixing with CT states, and therefore significantly deviates from the experimental data. This can be attributed to an overstabilization of CT states by the TD-DFT approach but does not mean that the almost iso-energetic CT state does not exist.^49^

### Folding-induced emission enhancement

**Fig. 7:**
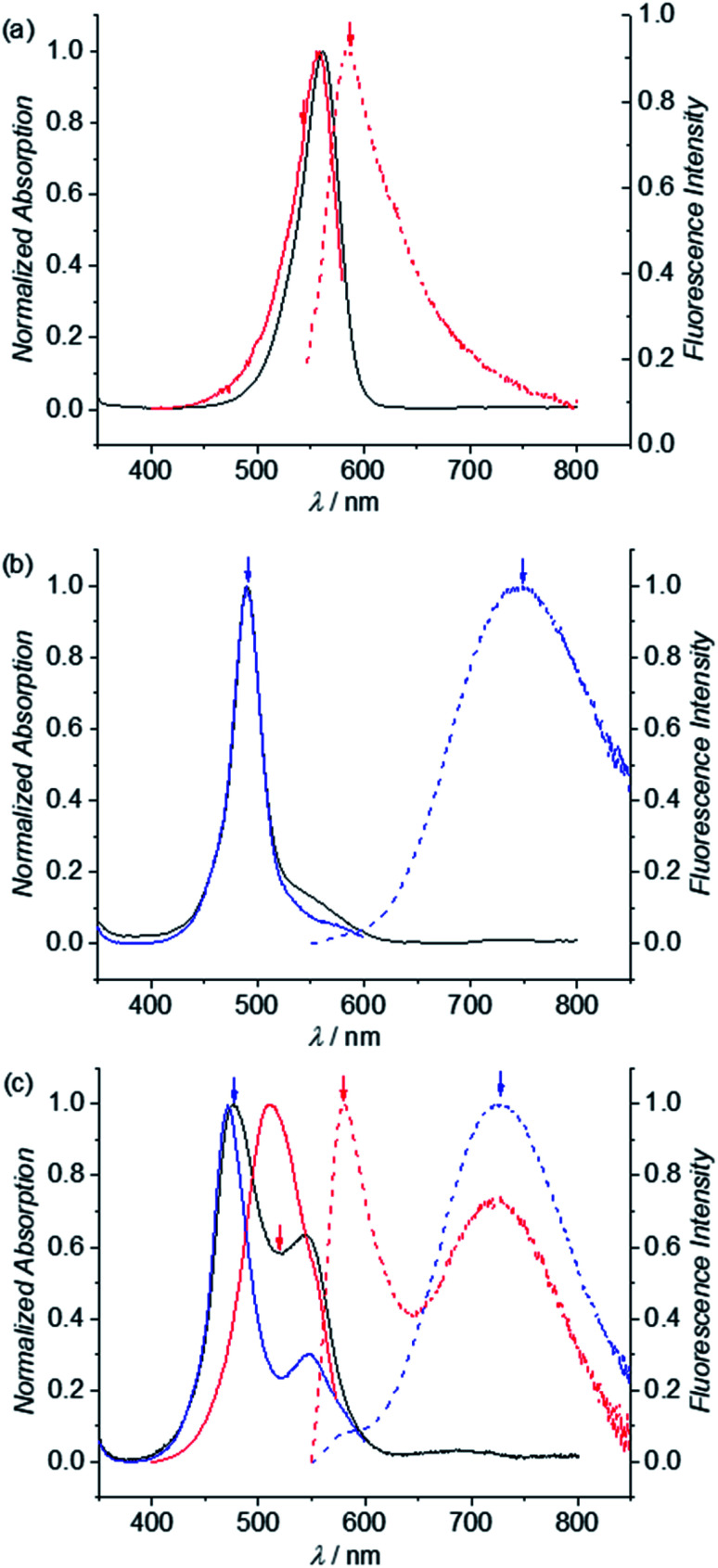
Normalized UV/vis absorption (black line), excitation (blue and red solid line) and emission spectra (blue and red dashed line) of reference merocyanine (a) **1**, (b) dimer **2** and (c) trimer **3** in CHCl_3_ (298 K, *c* = 3.5 × 10^−6^ M). Colored arrows indicate excitation or emission points of the corresponding emission or excitation spectra, respectively, with the same colors.

In our previous work we could show that the fluorescence of merocyanine dyes can enhance upon self-assembly due to the restriction of torsional motion in the aggregated state.^50,51^ Accordingly, such aggregation-induced enhanced emission (AIEE)^52^ was also expected to happen upon folding of MC dye oligomers **2–6**. As show in Fig. 7a, red dashed line, the emission spectrum of reference dye **1** in chloroform at low concentration (3.5 × 10^−6^ M) shows typical MC monomer emission properties, *i.e.* a sharp band at 584 nm with a small Stokes shift of 600 cm^−1^. In contrast, the emission spectrum of dimer **2** is significantly broadened and shows a large Stokes shift of 7000 cm^−1^, which is a characteristic feature of an excimer (Fig. 7b, blue dashed line).^53^ The excitation spectrum obtained for *λ*_em_ = 750 nm (Fig. 7, blue line) matches the absorption spectrum (black line), revealing that only one species is responsible for the emission. The emission spectrum of trimer **3** (*λ*_ex_ = 477 nm) likewise shows a typical excimer emission band located at 730 nm (Fig. 7c, blue dashed line). However, the respective excitation spectrum recorded for *λ*_em_ = 730 nm (blue solid line) shows significant deviations from the absorption spectrum (black line) indicating the presence of more than one emissive state. When excitation is performed at 520 nm, indeed an additional band is noted in the fluorescence spectrum at 580 nm, which can be attributed to the single merocyanine chromophore (red dashed line). This is further confirmed by the excitation spectra recorded for *λ*_em_ = 580 nm (red line), that closely resembles the monomer absorption spectrum (Fig. 7a). This supports our conclusion from UV/vis absorption spectroscopy on the coexistence of a triple stack causing the excimer emission band as well as a partially folded structure comprising a double stack and an electronically non-coupled single chromophore. The additional shoulder at 548 nm in the excitation spectrum for *λ*_em_ = 730 nm (blue line) might be due to an allowed transition to the lower exciton state of the triple stack.

The emission spectrum of tetramer **4** shows an excimer and a monomer emission band upon excitation at 470 nm as well (Fig. S14d,† blue dashed line). As expected, the intensity of the monomer band is increased compared to the excimer band (red dashed line) when increasing the excitation wavelength towards the monomer absorption (520 nm, red line), being highly indicative for an emission of the single chromophore at ∼576 nm. The fluorescence spectrum of pentamer **5** likewise shows two maxima at 576 and 730 nm, which can be assigned to the emission from a single chromophore and π-stacked MC chromophores, respectively (Fig. S14e,† blue dashed line). This is in agreement with the dependence of the emission band intensities on the excitation wavelength. Accordingly, upon excitation at 530 nm (*i.e.* the absorption maximum of the single chromophore), the fluorescence spectrum is dominated by the monomer emission band at 576 nm (Fig. S14e,† red dashed line), whereas excitation at 470 nm leads to a significant increase of the excimer emission band (Fig. S14e,† blue dashed line). The same behavior holds true for hexamer **6**: excitation at around 529 nm leads to a monomer-like emission with a dominant maximum at 575 nm (Fig. S14f,† red dashed line), while excitation at lower wavelengths significantly increases the intensity of the excimer-like band with a maximum at around 730 nm (Fig. S14f,† blue dashed line). Taken together, these experiments suggest a conformational heterogeneity for all larger oligomers beyond the dimer in chloroform solution that is also evident in the fluorescence decay. Thus, the photoexcited foldamer ensembles do not structurally relax within the lifetime of their excited states but decay depending differently on their original conformation in the ground state.

**Fig. 8:**
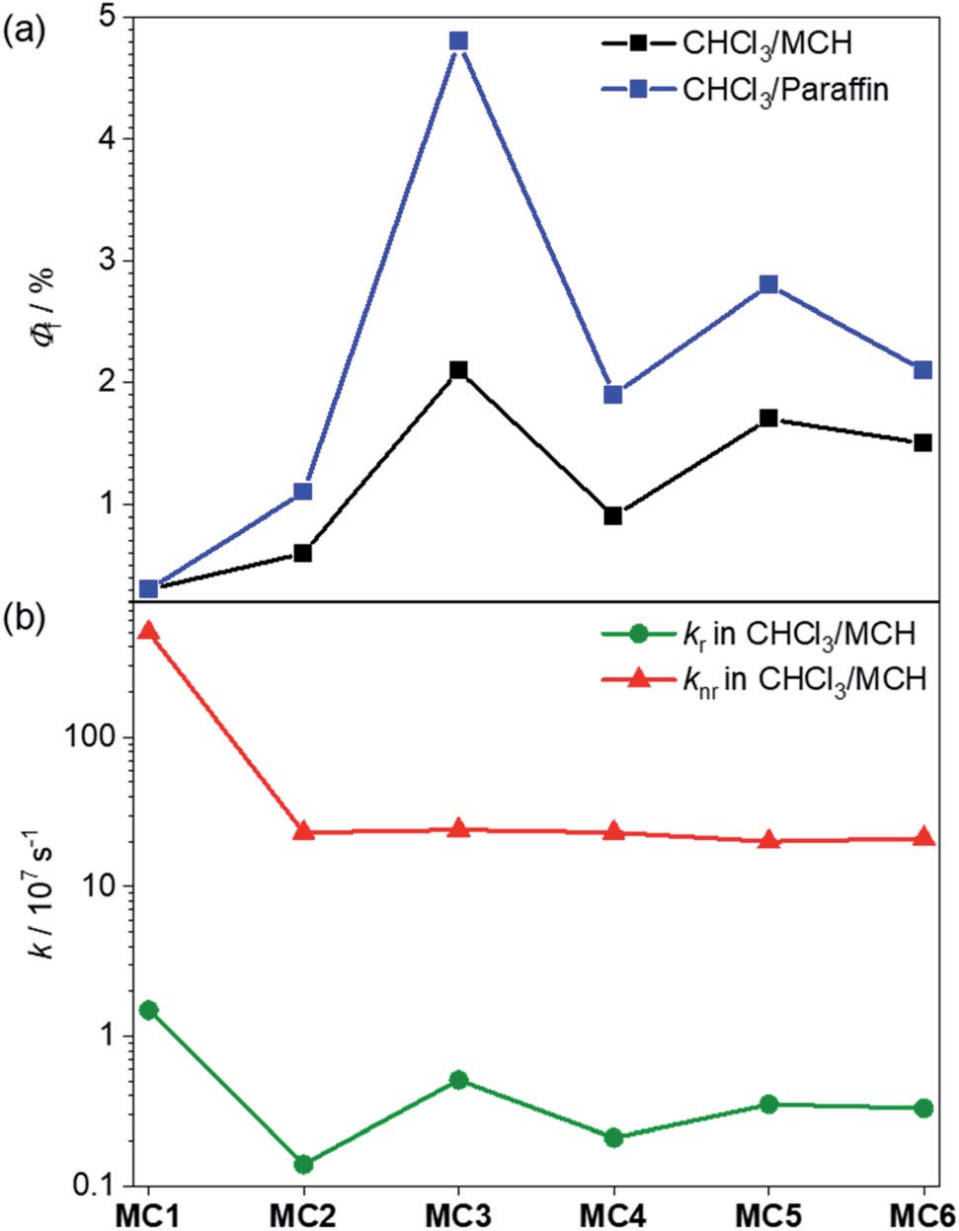
(a) Fluorescence quantum yields of reference MC **1** and MC oligomers **2–6** in CHCl_3_/MCH 30 : 70 (black) and CHCl_3_/liquid paraffin 30 : 70 (blue) at rt. (b) Calculated radiative (green) and non-radiative rate constants of MC **1–6** in CHCl_3_/MCH 30 : 70.

Fluorescence measurements were next performed in solvent mixtures of CHCl_3_/MCH 30 : 70 in order to investigate the excited state properties of the more tightly folded dye stacks. As expected, the fluorescence spectra of oligomers **2–6** display an excimer emission band at ∼730 nm upon excitation at the main absorption band (Fig. S15,† red lines). The excitation spectra are in good agreement with the respective UV/vis absorption spectra, revealing that now mainly one emissive state is present (Fig. S15,† blue lines). We have also determined the fluorescence quantum yields of the dye stacks in CHCl_3_/MCH 30 : 70. Accordingly, all folded oligomers **2–6** reveal a fluorescence enhancement in comparison to the monomeric reference dye with 0.3% (Table S2†). Interestingly, we obtained significantly higher fluorescence quantum yields for the odd-numbered trimer **3** (2.1%) and pentamer **5** (1.7%) compared to the even-numbered dimer **2** (0.6%) and tetramer **4** (0.9%) (Fig. 8, black). This AIEE result may at first glance surprise because of the optically forbidden transition from the lowest excited state to the ground state in these quite perfect H-aggregates.^37^ Indeed, the radiative rate constants calculated from the fluorescence lifetimes and quantum yields for this series of folded MC oligomers **2–6** show a decrease of about one order of magnitude (0.1–0.5 × 10^7^ s^−1^, Fig. 8b) compared to reference MC **1** (1.5 × 10^7^ s^−1^). However, concomitant with the stacking of the dyes, the common rapid non-radiative relaxation pathway for merocyanine dyes *via* a conical intersection upon rotation of the C–C bonds in the polymethine bridge^54,55^ is hindered. This is apparent from the non-radiative rate constant, which is significantly decreased for MC oligomers **2–6** (≈2 × 10^8^ s^−1^) compared to reference MC **1** (5 × 10^9^ s^−1^). While both rate constants are reduced in these MC oligomers, the change is more pronounced for the non-radiative rate constant, which obviously leads to the increase of the fluorescence quantum yield upon formation of the dye stacks in the folded state.

The observed odd–even effect for the fluorescence quantum yields (Fig. 8a) can be explained by the folding behavior. In the case of even-numbered oligomers **2** and **4**, MC_2_-units are predominant and therefore the similar fluorescence quantum yields and radiative and non-radiative decays are observed. In contrast, the formation of stacks with an odd number of chromophores results in higher fluorescence quantum yields with the highest value observed for the trimer **3**. Because all folded oligomers show very similar non-radiative rates between 20 × 10^7^ s^−1^ and 24 × 10^7^ s^−1^, *i.e.* are similarly “protected” against the non-radiative deactivation pathway *via* torsional motions toward a conical intersection, the odd–even effect is obviously attributable to differences in the radiative rate constants (Fig. 8b and Table S2†) which is higher for the odd-numbered MC oligomers and reaches its maximum for trimer **3**. This faster radiative relaxation is well understandable from the UV/vis absorption spectra and our quantum chemical calculations that revealed that the excitation into (and likewise emission from) the lowest excited state has a non-negligible oscillator strength for odd-membered MC stacks (Fig. 6). It is noteworthy that the fluorescence quantum yield could be further increased in a solvent mixture of CHCl_3_/liquid paraffin 30 : 70 (Fig. 8a, blue). Here, for the smaller MC oligomers **2–4** about a twofold enhancement of the emission was observed (Table S3†), with the fluorescence quantum yield for the trimer **3** rising from 2.1 to 4.8%. We attribute this effect to the higher viscosity of liquid paraffin, which is known to reduce molecular motions due to a more rigid environment (Table S3†).^56,57^ The increase in case of the larger MC oligomers **5** and **6** was smaller compared to oligomers **2–4**, presumably due to the lower folding tendency in paraffin (Fig. S18e and f†).

## Conclusion

In summary, this work explored the organization of merocyanine chromophores by the foldamer approach for a comprehensive series of increasing oligomer size from two up to six units. Our UV/vis spectroscopic investigations revealed that a hydrogen-bonded diamide pyridine turn moiety supported the folding for oligomers **2–6** by strong dipole–dipole interactions between the MC chromophores in low-polarity solvents. According to our studies, trimer **3** and pentamer **5** form triple and quintuple stacks in the non-polar solvent mixture, while tetramer **4** and hexamer **6** show only partial folding into two or three dimeric MC subunits, respectively. This is explained by the drastically reduced dipole–dipole interaction energy between folded MC_2_ pairs due to their vanished dipolarity in contrast to the single dipolar chromophore. Therefore, only MC oligomers containing an odd number of MC units undergo full folding into extended π-stacks beyond dimers. The folding process was confirmed by NMR spectroscopy in chloroform, where an NMR structure for trimer **3** showed a stack of three MC chromophores with perfect antiparallel orientation. The fluorescence quantum yield for all folded dye stacks is increased with respect to the single reference chromophore. This result is explained by our time-resolved fluorescence spectroscopy revealing a strong decrease of the non-radiative relaxation pathway due to more rigidified polymethine chains within the π-stacked folded state. Accordingly, for trimer **3** a 16-fold aggregation-induced emission enhancement could be observed in a viscous chloroform/paraffin solvent mixture compared to monomer **1**. In conclusion, our study could shine light on the hierarchical, size-dependent folding processes in oligomers of dipolar chromophores governed by dipole–dipole and dispersion interactions. In addition, we could show that the rigidification of non-fluorescent monomers in well-defined folded aggregate structures can lead to an increased luminescence even in the presence of strong H-type exciton coupling.

## Author contributions

F. W. conceived and supervised the project. X. H. and A. S. did the synthesis and spectroscopic studies. J. O. L. provided the quantum-chemical calculations. M. G. and D. B. helped with the evaluation of the NMR measurements and quantum chemical calculations, respectively. A. S. and F. W. wrote the manuscript with help from all authors.

## Conflicts of interest

There are no conflicts to declare.

## Supplementary Material

SC-012-D1SC01678D-s001
