# Supplementary material for: Folding and fluorescence enhancement with strong odd–even effect for a series of merocyanine dye oligomers
Source: Chem Sci. 2021 May 20;12(24):8342–52. doi: 10.1039/d1sc01678d (PMC8221066; doi:10.1039/d1sc01678d)
Supplement: SC-012-D1SC01678D-s001 [file SC-012-D1SC01678D-s001.pdf]

Electronic Supplementary Material (ESI) for Chemical Science.
This journal is © The Royal Society of Chemistry 2021

Electronic Supplementary Material (ESI)

Folding and fluorescence enhancement with strong odd-even effect for a series of merocyanine dye oligomers

Xiaobo Hu,[1] Alexander Schulz,[1] Joachim O. Lindner,[2] Matthias Grüne,[1] David Bialas,[2] and Frank Würthner[*,1,2]

[1] Institut für Organische Chemie, Universität Würzburg, Am Hubland, 97074 Würzburg, Germany
[2] Center for Nanosystems Chemistry (CNC), Universität Würzburg, Theodor-Boveri-Weg, 97074 Würzburg, Germany
[*] Email: wuerthner@uni-wuerzburg.de

Table of contents

Materials and methods ---------- S2
Synthesis and characterization ---------- S5
NMR studies ---------- S12
UV/Vis and fluorescence studies ---------- S23
Calculations---------- S30
1D NMR spectra ---------- S32
Mass spectra ---------- S37
Additional references ---------- S39

Materials and methods

All commercially available starting materials and reagents were used without further purification. Anhydrous DCM was dispensed from a solvent purification system. Analytical thin layer chromatography (TLC) was performed on silica gel plates (Merck 60F254) visualized with a UV lamp (254 nm). Column chromatography was performed with commercial glass columns using silica gel 60M (particle size 0.04-0.063 mm).Recycling gel permeation chromatography (GPC) was performed on a Shimadzu chromatography system with preparative JAIGEL columns (2 x 2H, 1 x 2.5H in a row). Melting points were determined with a BÜCHI Melting Point B-545 apparatus and are uncorrected.

UV/Vis spectroscopy

UV/Vis absorption spectra were recorded on a JASCO V670 or V770 spectrometer with a scan rate of 400 nm/min and a data interval of 0.5 nm. Conventional quartz cells from 0.01 mm to 100 mm path length were used to cover different concentrations throughout the study. Organic solvents for spectroscopic studies were of spectroscopic grade and used without further purification.

Fluorescence spectroscopy

Fluorescence spectra were recorded on an Edinburgh Instruments FLS 980 spectrometer with Xenon Xe1 lamp and visible PMT detector under following conditions: (1) for normal spectra, Dwell time = 0.2 s, step = 0.5 nm, number of scans = 10, ExBW ($\Delta\lambda$) = 4.0, EmBW ($\Delta\lambda$) = 4.0, and without polarizers; (2) for quantum yield determination, Dwell time = 1.0 s, step = 1.0 nm, number of scans = 10, ExBW ($\Delta\lambda$) = 6.0, EmBW ($\Delta\lambda$) = 6.0, ExPol = 0°, EmPol = 55°. For quantum yield determination, four emission spectra at four different excitation wavelengths were recorded for each sample and the given quantum yield represents the averaged value of four measurements. Quantum yield was determined against *N*,*N'*-bis(2,6-diisopropylphenyl)-1,6,7,12-tetraphenoxy-3,4:9,10-perylenetetracarboxylic diimide in $CHCl_3$ as the reference (Φ = 0.96[S1]) and the emission spectra of **2-5** were extrapolated using Gaussian functions before use (Fig. S16). The concentration of each sample was chosen by ensuring its UV/vis absorbance to be lower than 0.1(with 10 mm path length cell) to minimize re-absorption effect. Lifetime measurements were conducted with TCSPC diode and high speed lifetime PMT under following conditions: EmBW ($\Delta\lambda$) = 8.0, EmPol = 55°, 100 ns, 10000 peak counts. Organic

solvents for spectroscopic studies were of spectroscopic grade and used without further purification.

Mass spectrometry

High resolution electrospray ionization time-of-flight (HRESI-TOF) mass spectra were measured in the positive ion mode on a Bruker Daltonic microTOF-Q III spectrometer.

NMR spectroscopy

NMR spectra (^{1}H, ^{13}C, ^{1}H-^{1}H COSY, ^{1}H-^{1}H ROESY, and ^{1}H-^{1}H NOESY) were recorded on a Bruker Avance III HD 400 or Avance III HD 600 spectrometer in $CDCl_3$ at 295 K. A BBFO standard probe was used for 400 MHz and a DCH ^{13}C/^{1}H cryoprobe for 600 MHz, respectively. The residual solvent signals were used as internal standard (^{1}H: δ = 7.26 ppm, 13C: δ = 77.16 ppm) and the chemical shifts δ are reported in ppm. Abbreviations used for signal multiplicity are: s = singlet, d = doublet, t = triplet, q = quartet, m = multiplet or overlap of nonequivalent resonances, br = broad. Coupling constants, *J*, are reported in Hertz (Hz). Data processing was performed with the Topspin software.

Alternating-phase 180° pulses were applied during the mixing time of 300 ms in the ROESY pulse sequence (roesyphpp.2) to suppress unwanted TOCSY contributions.[S2, S3] This procedure is of particular importance, because the signal intensities of the 2D ROESY spectra were used for a quantitative evaluation.

Simulation of solution structures of 3 in chloroform based on 2D-NMR data

For simulating solution structures of **3** in chloroform, the relevant ROE correlations needed to be screened. Only correlations below the spectral diagonal were used and some redundant correlations were further deleted. Finally, twenty-five useful correlations were selected and converted into distance information via equations S1-S3[S4] (with the approximation that they are valid for the applied mixing sequence of consecutive 180°x/180°-x pulses as well) by using a fixed $6^A \leftrightarrow 5^A$ distance as the reference (Table S1). These distances were imported into an opened structure of **3** as distance constraints to perform energy minimization via MMFF in vacuum resulting in a folded structure with close anti-parallel organization of MC moieties, i.e.

MC_3 (Fig. S5). This process was repeated several times and always yielded similar structures (MC_3).

$$r_{ij} = r_{ref} \left(\frac{a_{ref} c_{ref}}{a_{ij} c_{ij}} \right)^{1/6} \qquad \text{(S1)}$$

$$c_{ij} = \frac{1}{(\sin^2 \theta_i \sin^2 \theta_j)} \qquad \text{(S2)}$$

$$\tan \theta_i = \frac{\gamma B_1}{(\omega_i - \omega_0)} \qquad \text{(S3)}$$

Computational details

For the simulation of UV/vis spectra, all structures were optimized in the frame of density functional theory (DFT) using the B3LYP functional[S5] together with the def2-SVP basis set[S6] in TURBOMOLE[S7]. The 10 lowest excited states were calculated for every molecule using time-dependent density functional theory (TD-DFT) in Gaussian16[S8]. Correct long-range behavior of the method was ensured by using the range-separated hybrid functional CAM-B3LYP[S9] together with the same basis set as described above. Broadened spectra were obtained by convolution with Gaussians of 0.2 eV width. In order to compensate the overestimation of transition energies by the employed functional, a static shift of 0.491 eV to lower energies was applied to all calculated TD-DFT spectra. In all DFT calculations, Grimme's empirical D3 correction[S10] was used to account for the correct dispersion interactions.

Absorption spectra on the basis of Kasha's molecular exciton theory were calculated based on the monomer absorption at 560 nm with a calculated oscillator strength of 1.4753 on the TD-DFT level of theory as described above. Coupling constants of 0.12 eV and 0.18 eV were used in the case of **2** and **3**, respectively. Additionally, the effects of van der Waals stabilization in the excited states as well as solvatochromism were compensated by a static shift of 0.03 eV (0.09 eV) to lower energies for molecule **2** (**3**).

Synthesis and characterization

The syntheses of precursors **7-12** and pentamer **5** are described in our earlier work.[S11]

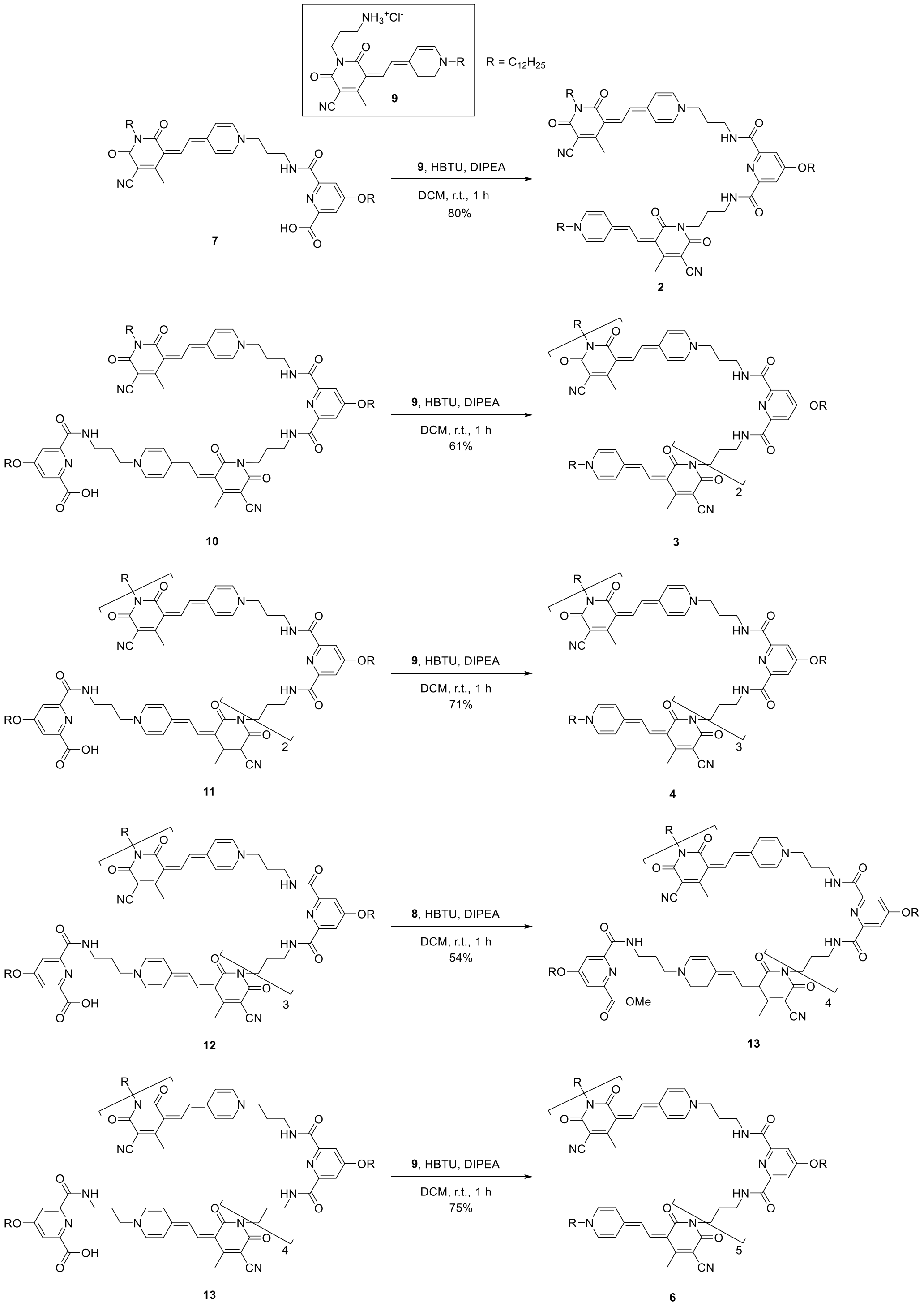

Scheme S1 Synthesis of merocyanine oligomers **2-6**. HBTU = (2-(*1H*-benzotriazol-1-yl)-1,1,3,3-tetramethyluronium hexafluorophosphate, DIPEA = *N*,*N*-diisopropylethylamine.

Synthesis of dimer **2**

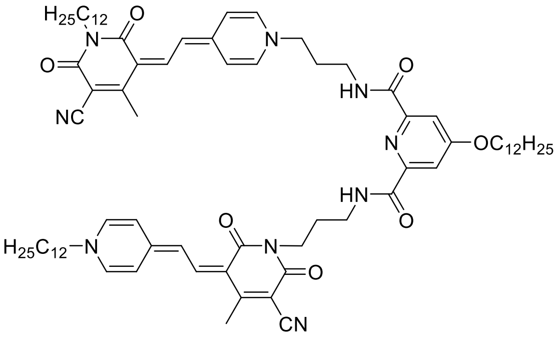

2

To a suspension of compound**7** (16 mg, 20 μmol, 1 equiv.), compound **9** (14 mg, 30 μmol, 1.5 equiv.) and HBTU (15 mg, 40 μmol, 2 equiv.) in 2 mL anhydrous dichloromethane, DIPEA (18μL, 0.10 mmol, 5 equiv.) was added. The reaction mixture was stirred at room temperature for 1 h under nitrogen atmosphere. The solvent was removed under vacuum and the crude product washed with methanol (3 x 10 mL) and then purified by column chromatography (eluent: DCM/MeOH = 94/6, v/v) followed by GPC to yield the dimer **2** as a dark red solid (20 mg, 16 μmol, 80%). **Mp.**: 235–238 °C (from CH_2Cl_2). **^{1}H NMR** (400 MHz, $CDCl_3$): δ (ppm) = 9.67 (t, *J* = 6.7 Hz, 1H), 9.45 (t, *J* = 5.6 Hz, 1H), 7.95–7.72 (m, 6H), 7.69 (d, *J* = 6.5 Hz, 2H), 7.39 (d, *J* = 15.2 Hz, 1H), 7.33 (d, *J* = 14.8 Hz, 1H), 7.27 (m, overlapped with C*H*Cl_3 signal) 7.18 (d, *J* = 7.0 Hz, 2H), 4.41 - 4.19 (m, 6H), 4.14 (t, *J* = 6.5 Hz, 2H), 3.99–3.92 (m, 2H), 3.80 (br, 4H), 2.63–2.53 (m, 2H), 2.30 (s, 3H), 2.19 (s, 3H), 2.06–1.92 (m, 4H), 1.87–1.79 (m, 2H), 1.66–1.58 (m, overlapped with H_2O signal), 1.49–1.15 (m, 54H), 0.90–0.79 (m, 9H).**^{13}C NMR** (101 MHz, $CDCl_3$): δ (ppm) = 168.2, 165.3, 164.6, 163.6, 163.5, 163.3, 163.1, 156.9, 156.72, 156.70, 156.0, 151.0, 150.1, 140.9, 139.7, 139.5, 138.7, 120.4, 119.6, 119.3, 114.8, 113.6, 110.6, 106.4, 106.3, 88.7, 87.3, 69.1, 60.4, 55.5, 39.9, 36.6, 35.7, 34.5, 32.1, 32.0, 30.9, 29.9, 29.83, 29.80, 29.77, 29.72, 29.68, 29.6, 29.51, 29.49, 29.47, 29.4, 29.1, 28.9, 28.6, 27.5, 27.0, 26.4, 26.0, 22.8, 18.9, 18.7, 14.3.**HRMS** (ESI, positive mode): *m/z* calcd for $C_{77}H_{109}N_9NaO_7$ [M + Na]$^+$: 1294.8342, found: 1294.8334.UV/Vis ($CHCl_3$, c = 4 x 10^{-6} M): λ_{max} = 490 nm (ε = 213000 $M^{-1}cm^{-1}$).

Synthesis of trimer **3**

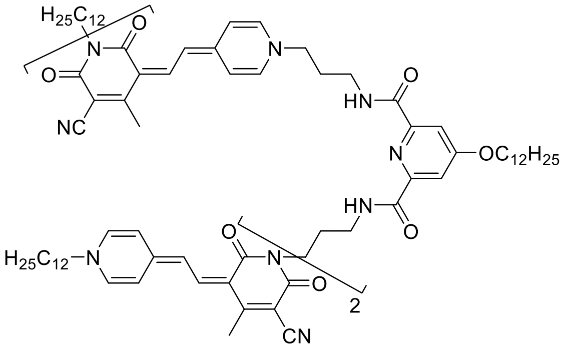

3

To a suspension of compound**10** (15 mg, 10 μmol, 1 equiv.), compound **9** (7.0 mg, 15 μmol, 1.5 equiv.) and HBTU (11 mg, 30 μmol, 3 equiv.) in 2 mL anhydrous dichloromethane, DIPEA (10 μL, 60 μmol, 6 equiv.) was added. The reaction mixture was stirred at room temperature for 1 h under nitrogen atmosphere. The solvent was removed under reduced pressure and the crude product washed with methanol (3 x 10 mL) and further purified by column chromatography (eluent: DCM/MeOH = 94/6,v/v) followed by GPC to yield trimer **3** as a dark red solid (12 mg, 6.1 μmol, 61%). **Mp.**: 166–168 °C (from CH_2Cl_2).**^{1}H NMR** (400 MHz, $CDCl_3$): δ (ppm) = 9.80 (t, J = 6.4 Hz, 1H), 9.67 (t, J = 6.5 Hz, 1H), 9.55 (t, J = 5.9 Hz, 1H), 9.42 (t, J = 6.0 Hz, 1H), 8.01 (d, J = 6.6 Hz, 2H), 7.88 (d, J = 14.7 Hz, 1H), 7.82–7.80 (m, 2H), 7.78–7.72 (m, 5H), 7.71–7.65 (m, 3H), 7.58 (d, J = 15.0 Hz, 1H), 7.41–7.33 (m, 3H), 7.28 (m, overlapped with C*H*Cl_3 signal), 7.22 (d, J = 6.7 Hz, 2H), 7.15 (d, J = 6.7 Hz, 2H), 4.48–4.36 (m, 2H), 4.32–4.07 (m, 10H), 4.03–3.97 (m, 2H), 3.93–3.85 (m, 2H), 3.79–3.69 (m, 4H), 3.31 (br, too broad to be integrated), 2.55–2.44 (m, 4H), 2.38 (s, 3H), 2.25–2.19 (m, 6H), 2.08–1.96 (br, 4H), 1.88–1.71 (m, 6H), 1.72–1.57 (m, overlapped with H_2O signal), 1.50–1.42 (m, 4H), 1.36–1.08 (m, 64H), 0.91–0.79 (m, 12H). **^{13}C NMR** (101 MHz, $CDCl_3$): δ (ppm) = 168.3, 168.2, 165.1, 165.0, 164.72, 164.65, 163.7, 163.4, 163.3, 163.0, 157.2, 156.9, 156.7, 156.5, 156.3, 156.0, 151.0, 150.3, 150.2, 141.4, 140.4, 140.0, 139.7, 139.6, 137.6, 120.8, 120.6, 120.0, 119.6, 119.3, 115.4, 114.6, 113.3, 110.9, 110.8, 110.7, 110.6, 106.9, 106.5, 106.4, 89.0, 87.9, 86.2, 69.2, 69.1, 59.8, 57.3, 55.8, 39.9, 36.83, 36.76, 36.3, 35.9, 34.6, 34.5, 32.1, 32.0, 31.4, 31.0, 29.9, 29.83, 29.79, 29.77, 29.72, 29.69, 29.6, 29.51, 29.49, 29.47, 29.45, 29.0, 28.9, 28.4, 28.1, 27.53, 27.45, 26.2, 26.0, 22.8, 18.84, 18.82, 18.7, 14.3.**HRMS** (ESI, positive mode): *m/z* calcd for $C_{116}H_{159}N_{15}NaO_{12}$ $[M + Na]^+$: 1977.2185, found: 1977.2187.UV/Vis ($CHCl_3$, c = 4 x 10^{-6} M): λ_{max} = 476 nm (ε = 137000 $M^{-1}cm^{-1}$).

Synthesis oftetramer**4**

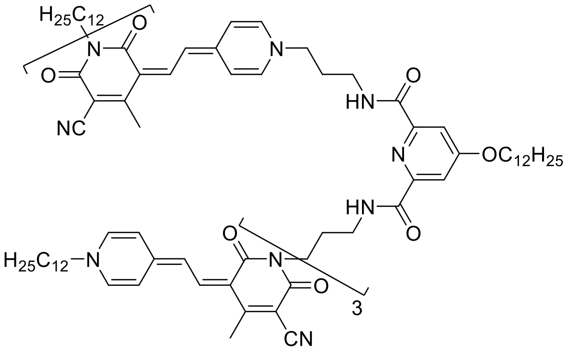

4

To a suspension of compound**11** (16 mg, 7.5 μmol, 1 equiv.), compound **9** (7.2 mg, 15 μmol, 2 equiv.) and HBTU (8.7 mg, 23 μmol, 3 equiv.) in 2 mL anhydrous dichloromethane, DIPEA (8.0 μL, 45 μmol, 6 equiv.) was added. The reaction mixture was stirred at room temperature for 1 h under nitrogen atmosphere. The solvent was removed under reduced pressure and the crude product washed with methanol (3 x 10 mL) and further purified by column chromatography (eluent: DCM/MeOH = 94/6,v/v) followed by GPC to yieldtetramer **4**as a dark red solid (14 mg,5.3 μmol, 71%). **Mp.**: 214–217 °C (from CH_2Cl_2). **^{1}H NMR** (400 MHz, $CDCl_3$): δ (ppm) = 9.79 (t, J = 6.5 Hz, 1H), 9.70–9.63 (m, 2H), 9.54 (t, J = 6.6 Hz, 1H), 9.45 (t, J = 6.3 Hz, 1H), 9.40 (t, J = 6.1 Hz, 1H), 8.06–7.95 (m, 6H), 7.93 (d, J = 14.9 Hz, 1H), 7.89–7.68 (m, 11H), 7.56 (d, J = 14.6 Hz, 1H), 7.51–7.41 (m, 5H), 7.35 (d, J = 6.8 Hz, 2H), 7.31 (d, J = 6.7 Hz, 2H), 7.23 (d, J = 6.9 Hz, 2H), 4.41–4.04 (m, 20H), 3.95–3.87 (m, 2H), 3.78–3.67 (m, 6H), 3.40 (br, too broad to be integrated), 2.58–2.43 (m, 6H), 2.37 (s, 3H), 2.36 (s, 3H), 2.35 (s, 3H), 2.30 (s, 3H), 2.13–1.93 (br, 6H), 1.88–1.72 (m, 8H), 1.66–1.56 (m, overlapped with H_2O signal), 1.50–1.41 (m, 6H), 1.38–1.14 (m, 80H), 0.91–0.83 (m, 15H).**^{13}C NMR** (151 MHz, $CDCl_3$): δ (ppm) = 168.3, 168.2, 165.23, 165.20, 164.88, 164.86, 164.84, 164.75, 163.7, 163.6, 163.5, 163.43, 163.36, 163.3, 163.2, 157.1, 157.02, 156.97, 156.8, 156.54, 156.49, 156.3, 156.1, 151.03, 151.02, 150.2, 150.1, 150.0, 141.3, 141.2, 141.0, 140.0, 139.8, 138.81, 138.77, 138.4, 128.6, 128.5, 126.0, 121.2, 120.9, 120.7, 120.14, 120.11, 120.0, 119.8, 115.2, 115.0, 113.5, 110.9, 110.7, 110.5, 106.8, 106.7, 106.6, 106.5, 88.4, 86.9, 86.84, 86.76, 69.2, 69.1, 59.8, 57.5, 57.0, 56.2, 39.9, 38.1, 36.9, 36.8, 36.7, 36.4, 36.1, 34.8, 34.7, 34.6, 32.07, 32.05, 32.0, 31.2, 30.5, 29.91, 29.86, 29.84, 29.81, 29.79, 29.77, 29.73, 29.72, 29.69, 29.68, 29.6, 29.52, 29.49, 29.46, 29.4, 29.1, 29.0, 28.9, 28.5, 27.6, 27.52, 27.45, 27.4, 26.2, 26.01, 25.99, 22.83, 22.81, 18.9, 18.84, 18.76, 18.7, 14.27, 14.26. **HRMS** (ESI, positive mode): m/z calcd for $C_{155}H_{209}N_{21}O_{17}$ $[M]^{2+}$: 1318.3062, found: 1318.3047.UV/Vis ($CHCl_3$, c = $4 \cdot 10^{-6}$ M): λ_{max} = 498 nm (ε = 215000 $M^{-1}cm^{-1}$).

Synthesis of **13**

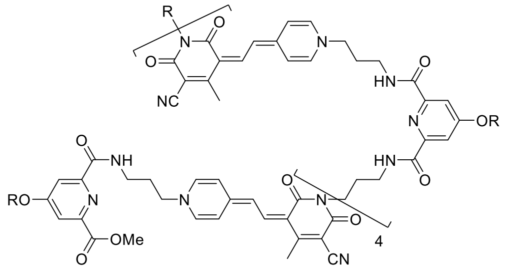

13

To a suspension of compound 1**2** (23 mg, 8 µmol, 0.8 equiv.), compound **8** (8.2 mg, 10 µmol, 1 equiv.) and HBTU (7.6 mg, 20 µmol, 2 equiv.) in 2 mL anhydrous dichloromethane, DIPEA (7.0 µL, 40 µmol, 4 equiv.) was added. The reaction mixture was stirred at room temperature for 1 h under nitrogen atmosphere. The crude product was washed with 10 mL methanol three times and further purified by column chromatography (eluent: DCM/MeOH =94/6, v/v%) to yield compound **13**as a dark red solid (15 mg, 5.4 µmol,54%). **Mp.**: 195–197 °C (from CH_2Cl_2). **^{1}H NMR** (400 MHz, $CDCl_3$): δ (ppm) = 9.77–9.67 (m, 4H), 9.49–9.36 (m,4H), 8.59 (t, *J* = 6.4 Hz, 1H), 8.23 (d, *J* = 6.8 Hz, 1H), 8.19–8.10 (m, 6H), 7.91–7.67 (m, 18H), 7.61–7.49 (m, 4H), 7.48–7.36 (m, 6H), 7.31 (d, *J* = 6.7 Hz, 2H), 7.23 (d, *J* = 6.7 Hz, 2H), 4.45–4.30 (m, 8H), 4.30–4.16 (m, 10H), 4.14–4.06 (m, 10H),3.98 (s, 3H), 3.92 (t,*J* = 7.3 Hz, 2H), 3.76–3.68(m, 6H), 3.59–3.52 (m, 2H), 3.40 (br, too broad to be integrated), 2.51–2.42 (m, 16H), 2.36 (s, 3H), 2.31–2.24 (m, 5H), 2.03–1.90 (m, 12H), 1.85–1.76 (m, 10H), 1.61–1.53 (m, 2H), 1.47–1.40 (m, 10H), 1.34–1.18 (m, 96H), 0.89–0.82 (m, 18H). **^{13}C NMR** (101 MHz, $CDCl_3$): δ (ppm) = 168.2, 167.8, 165.1, 165.0, 164.9, 164.8, 163.73, 163.70, 163.6, 163.5, 163.39, 163.35, 163.2, 157.4, 157.2, 157.1, 157.0 , 156.7, 156.5, 156.41, 156.36, 155.9, 151.4, 151.0, 150.0, 149.92, 149.89, 148.1, 141.4, 141.3, 141.2, 140.1, 139.8, 139.6, 139.1, 138.8, 138.4, 121.1, 121.0, 120.9, 120.7, 120.5, 120.3, 120.1, 119.7, 115.1, 115.0, 113.5, 111.1, 110.7, 110.5, 107.0, 106.8, 106.6, 106.5, 88.45, 87.05, 86.90, 86.53, 86.49, 77.36, 69.36, 69.10, 57.70, 57.05, 56.42, 53.57, 53.18, 50.95, 39.98, 36.84, 36.25, 35.85, 34.75, 32.03, 31.70, 30.53, 30.36, 29.86, 29.81, 29.77, 29.75, 29.70, 29.67, 29.47, 29.43, 29.39, 29.10, 28.92, 28.86, 28.43, 27.56, 25.97, 22.81, 18.81, 18.72, 14.26 **HRMS** (ESI, positive mode): *m/z* calcd for $C_{205}H_{271}N_{29}Na_2O_{26}$ $[M+2Na]^{2+}$: 1800.5280, found: 1800.5225.

Deprotection of **13** to **13-OH**

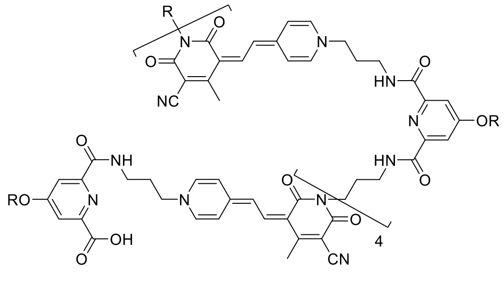

13-OH

13 (15 mg, 4.2 μmol, 1 equiv.) was dissolved in 2 mL MeOH/THF (30/70, v/v%) and 0.3 M NaOH aqueous solution (42 μL, 12.6 μmol, 3 equiv.) was added into the solution at room temperature. The mixture was stirred at room temperature for 2 h (note: the desired product slowly decomposes, therefore, the appropriate reaction time should be determined by TLC monitoring). The reaction mixture was then diluted with DCM and extracted with 10 mM HCl aqueous solution three times. The organic layer was dried with Na_2SO_4 and condensed under vacuum to yield a dark red solid. This crude product was rigorously dried under high vacuum and used for the next reaction without further purification.

Synthesis of hexamer **6**

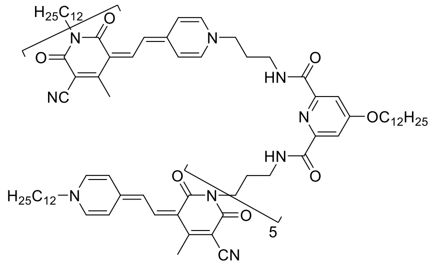

6

To a suspension of compound**13-OH** (11 mg, 3.1 μmol, 1 equiv.), compound **9** (2.3 mg, 4.5 μmol, 1.5 equiv.) and HBTU (3.4 mg, 9 μmol, 3 equiv.) in 2 mL anhydrous dichloromethane, DIPEA (2.5 μL, 15 μmol, 5 equiv.) was added. The reaction mixture was stirred at room temperature for 1 h under nitrogen atmosphere. The solvent was removed under reduced pressure and the crude product washed with methanol (3 x 10 mL) and further purified by column chromatography (eluent: DCM/MeOH = 94/6,v/v) followed by GPC to yield hexamer**6** as a dark red solid (9 mg, 2.3μmol,75%). **Mp.**: 241–245 °C (from CH_2Cl_2).**^{1}H NMR** (400 MHz, $CDCl_3$): δ (ppm) = 9.81–9.64 (m, 5H), 9.52–9.41 (m, 4H), 9.37 (t, J = 5.7 Hz, 1H), 8.19–8.10 (m, 8H), 7.97–7.88 (m, 3H), 7.86–7.76 (m, 6H), 7.75–7.71 (m, 12H), 7.60–7.53 (m, 3H), 7.51–7.47 (br, 2H), 7.46–7.37 (m, 8H), 7.30 (d, J = 6.1 Hz, 2H), 7.22 (d, J = 6.5 Hz, 2H), 4.44–4.33 (m, 8H), 4.28–4.15 (m, 12H), 4.12–4.08 (m, 10H), 3.92 (t,J = 7.6 Hz, 2H), 3.76–3.68(m, 10H), 3.36 (br, too broad to be integrated), 2.60–2.42 (m, 20H), 2.36 (s, 3H), 2.29 (s, 3H), 2.04–1.97 (br, 8H), 1.84–1.78 (m, 10H), 1.70–1.65 (m, 4H), 1.64 (m, 2H), 1.47–1.42 (m, 8H), 1.41–1.21 (m, 120H), 0.90–0.79 (m, 21H).**^{13}C NMR** (101 MHz, $CDCl_3$): δ (ppm) = 168.22, 168.18, 165.1, 165.00, 164.97, 164.91, 164.88, 164.8, 163.8, 163.7, 163.6, 163.5, 163.4, 163.39, 163.35, 163.2, 157.3, 157.1, 157.0, 156.8, 156.5, 156.42, 156.35, 156.0,

151.0, 150.06, 150.04, 149.99, 149.93, 149.89, 141.4, 141.3, 141.2, 140.8, 140.1, 139.8, 139.6, 139.2, 138.9, 138.4, 135.3, 125.1, 121.2, 121.0, 120.7, 120.58, 120.52, 120.39, 120.30, 120.22, 119.7, 115.1, 115.0, 113.5, 110.79, 110.73, 110.66, 110.52, 110.48, 107.0, 106.84, 106.83, 106.80, 106.6, 106.5, 88.54, 87.16, 86.89, 86.64, 86.60, 86.57, 69.18, 69.13, 59.95, 57.67, 57.60, 56.36, 53.58, 39.96, 36.86, 36.73, 36.21, 34.78, 34.68, 32.33, 32.05, 32.02, 31.93, 31.29, 30.51, 30.45, 30.16, 29.89, 29.84, 29.83, 29.79, 29.77, 29.72, 29.69, 29.60, 29.51, 29.48, 29.46, 29.45, 29.09, 28.93, 28.47, 27.59, 27.51, 26.52, 26.31, 25.99, 23.57, 22.83, 22.81, 18.90, 18.86, 18.85, 18.83, 18.75, 14.27. **HRMS** (ESI, positive mode): *m/z* calcd for $C_{233}H_{309}N_{33}Na_3O_{27}$ $[M+3Na]^{3+}$: 1356.7832, found: 1356.7849. UV/Vis ($CHCl_3$, $c = 4 \cdot 10^{-6}$ M): λ_{max} = 529 nm (ε = 315000 $M^{-1}cm^{-1}$).

NMR studies

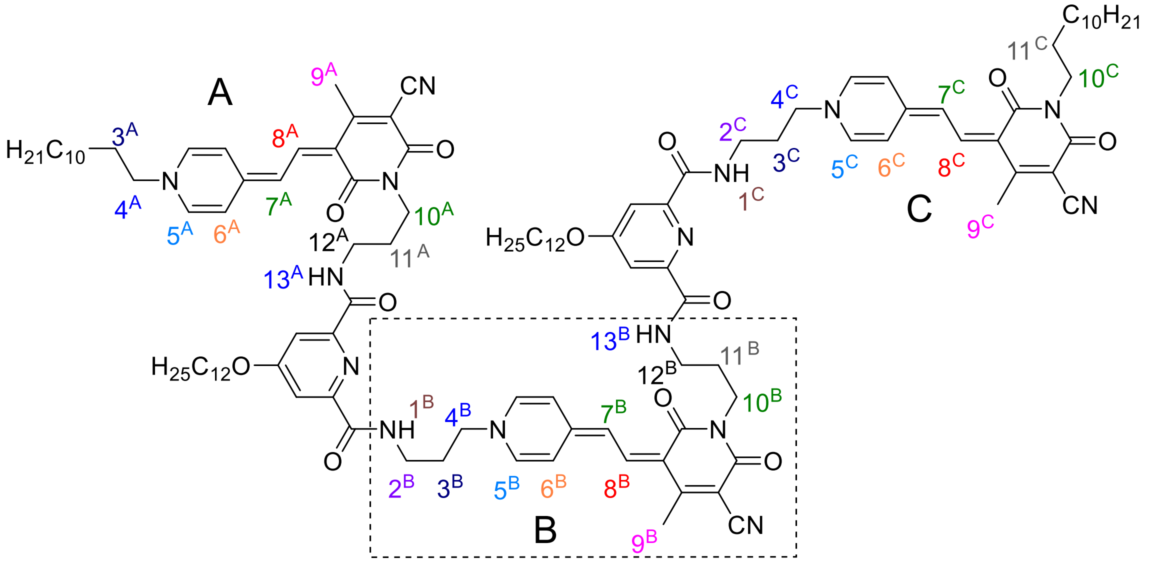

Fig. S1 Chemical structure of trimer **3** with the assignment of the protons used for the evaluation of the NMR studies. The different chromophores are labeled as A, B and C.

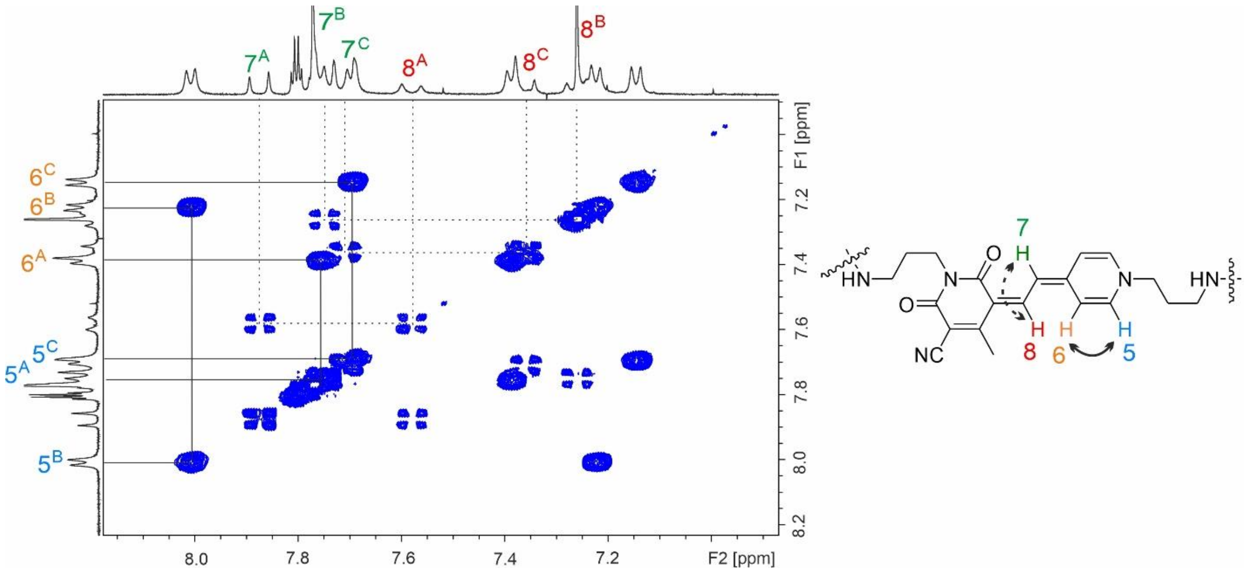

Fig. S2 Excerpt of ^1H-^1H COSY spectrum (400 MHz) of trimer **3** in $CDCl_3$ at 295 K showing 7 ↔ 8 correlations (dashed double-headed arrows) and 5 ↔ 6 correlations (solid double-headed arrows) in the same spin systems.

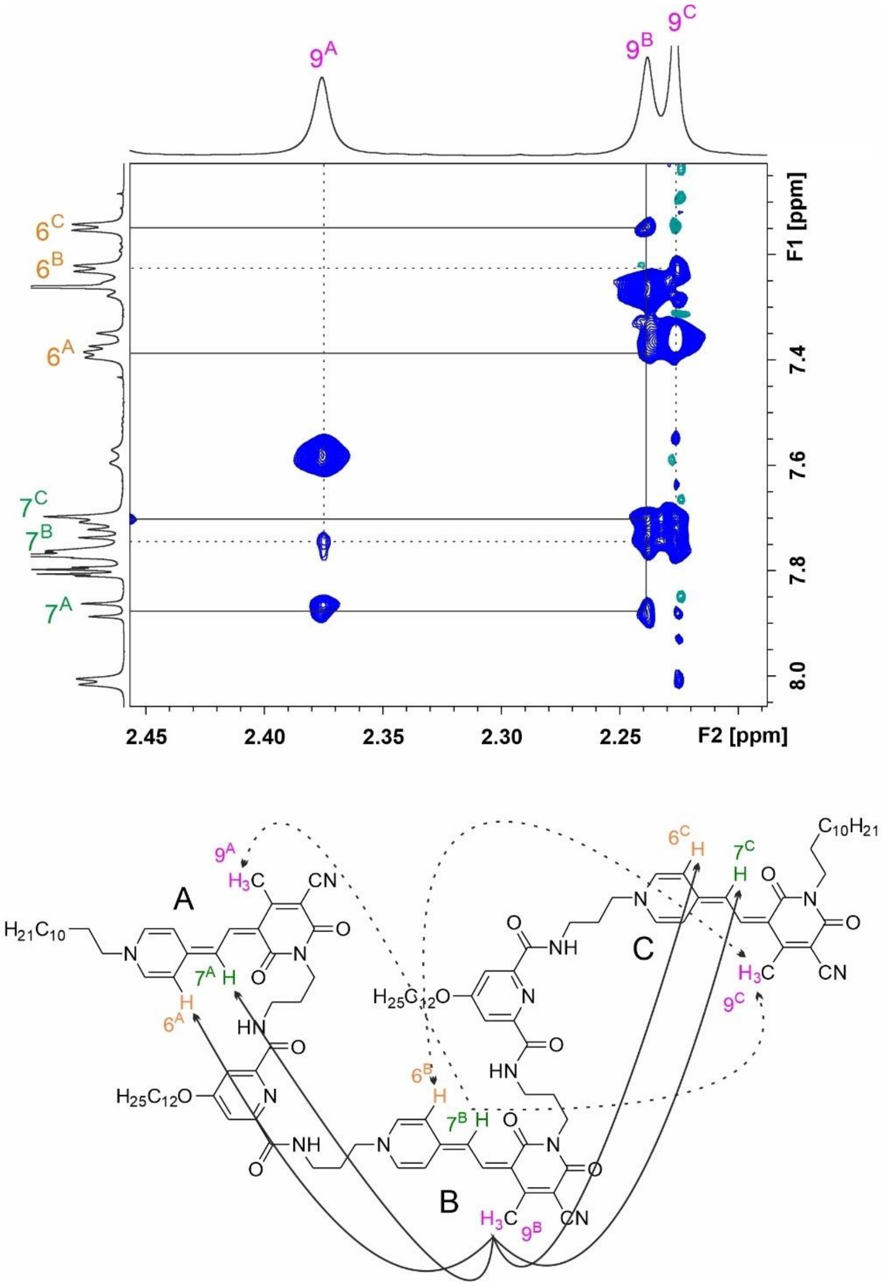

Fig. S3 Excerpt of the ^1H-^1H ROESY spectrum (600 MHz) of trimer **3** in $CDCl_3$ at 295 K. Also shown is the chemical structure of **3** with significant correlations between protons indicated by double-headed arrows.

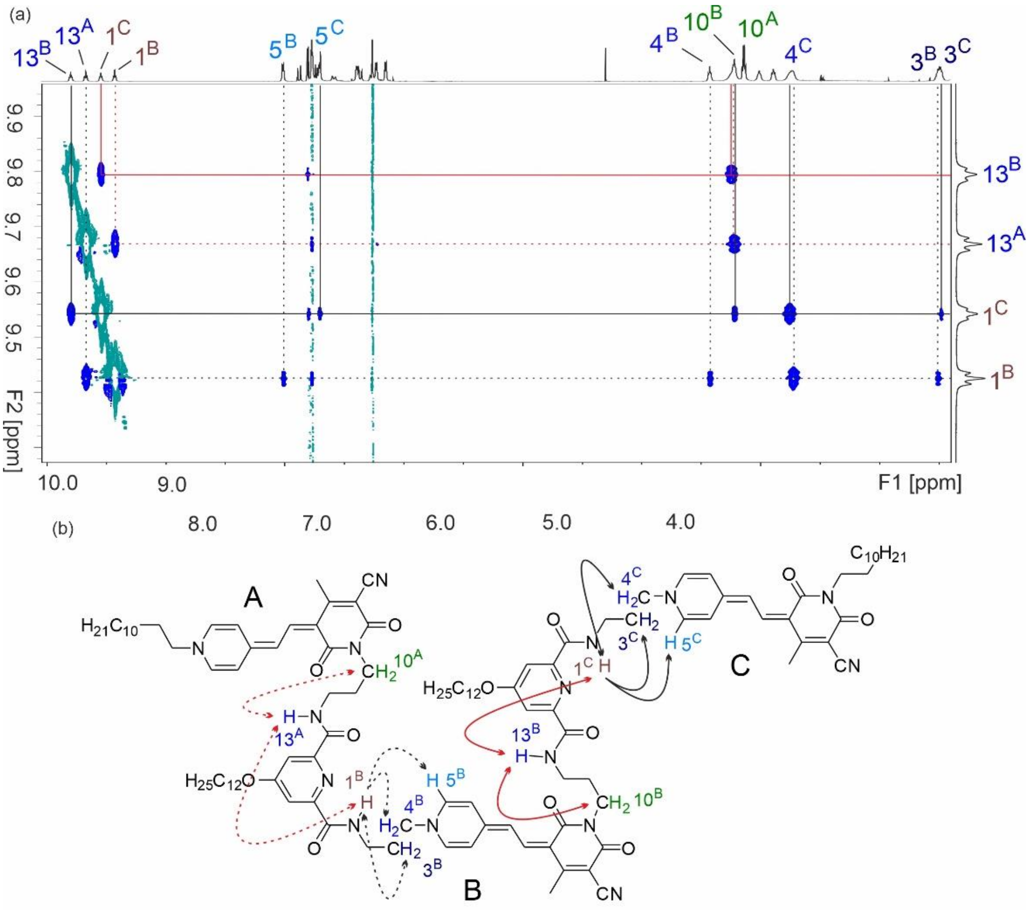

Fig. S4 An excerpt of the ^{1}H-^{1}H ROESY spectrum (600 MHz) of **3** in $CDCl_3$ at 295 K. Also shown is the chemical structure of trimer **3** with the correlations indicated by double-headed arrows as found in the ^{1}H-^{1}H ROESY spectrum.

Table S1 Selected ROE correlations and the corresponding distances in trimer **3**.

ROE correlations[a]	F2 (ppm)	F1 (ppm)	Distances r_{ij} (Å)[b]
$9^A \leftrightarrow 7^B$	2.37	7.75	3.74
$9^B \leftrightarrow 7^A$	2.24	7.88	3.64
$9^B \leftrightarrow 7^C$	2.24	7.70	3.27
$9^B \leftrightarrow 6^A$	2.24	7.38	3.63
$9^B \leftrightarrow 6^C$	2.24	7.15	3.91
$9^C \leftrightarrow 7^B$	2.23	7.75	3.17
$9^C \leftrightarrow 6^B$	2.23	7.23	3.46
$1^C \leftrightarrow 13^B$	9.54	9.79	2.60
$1^B \leftrightarrow 13^A$	9.42	9.67	2.31
$5^B \leftrightarrow 13^A$	8.01	9.67	4.15
$5^B \leftrightarrow 1^B$	8.01	9.42	3.18
$5^C \leftrightarrow 1^C$	7.70	9.54	3.55
$4^B \leftrightarrow 1^B$	4.42	9.42	3.35
$10^B \leftrightarrow 13^B$	4.24	9.79	2.80
$10^A \leftrightarrow 13^A$	4.23	9.67	2.77
$4^C \leftrightarrow 1^C$	4.22	9.54	3.27
$2^C \leftrightarrow 1^C$	3.75	9.54	2.62
$2^B \leftrightarrow 1^B$	3.72	9.42	2.43
$3^B \leftrightarrow 1^B$	2.51	9.42	3.43
$3^C \leftrightarrow 1^C$	2.48	9.54	3.69
$10^A \leftrightarrow 5^B$	3.89	8.01	3.57
$2^C \leftrightarrow 5^C$	3.76	7.70	3.30
$2^B \leftrightarrow 5^B$	3.72	8.01	3.19
$3^B \leftrightarrow 5^B$	2.51	8.01	2.61
$3^C \leftrightarrow 5^C$	2.48	7.70	2.55
Reference ($6^A \leftrightarrow 5^A$)	7.39	7.76	2.30[c]

[a] Selected from ^{1}H-^{1}H ROESY spectrum (600 MHz, 295 K) of **3** in $CHCl_3$.[b] Calculated using the equations [S1-S3]: $r_{ij} = r_{ref}\left(\frac{a_{ref}c_{ref}}{a_{ij}c_{ij}}\right)^{1/6}$; $c_{ij} = \frac{1}{(\sin^2\theta_i \sin^2\theta_j)}$; $\tan\theta_i = \frac{\gamma B_1}{(\omega_i - \omega_0)}$. [c] An approximate value from a crystal structure of a monomeric analogue with the same merocyanine moiety.

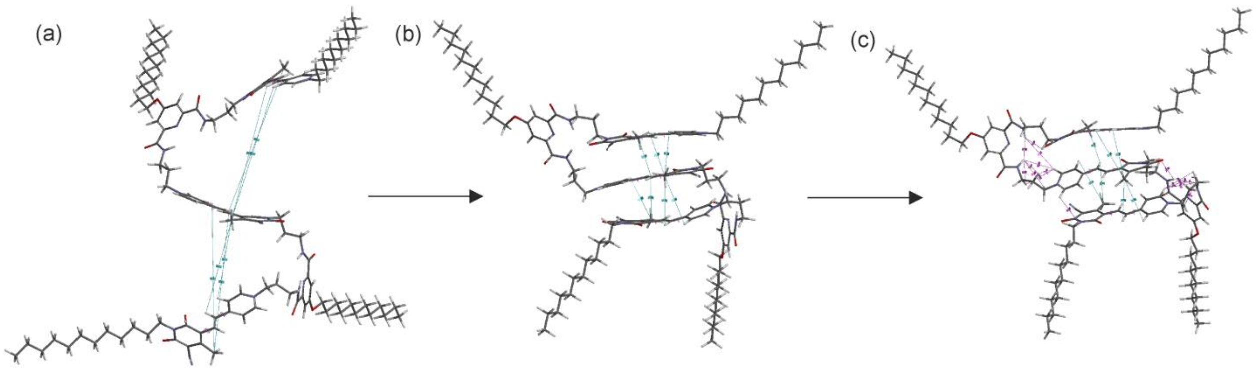

Fig. S5 Molecular modeling of trimer **3** with distance constraints (obtained from ROE correlations, Table S1) by Spartan via MMFF (energy minimization in vacuum). (a) The starting opened structure, (b) the resulting folded structure (H-aggregate) after putting partial distance constraints (only those proton 9 related correlations, blue lines) and (c) the final close anti-parallel stack after putting all distance constraints (blue and violet lines).

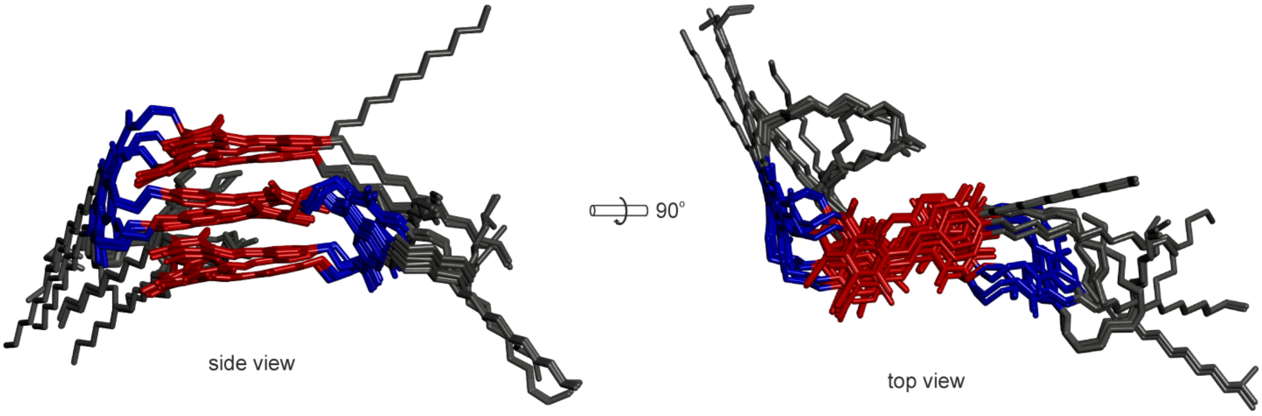

Fig. S6 Overlapped fourteen conformers of trimer **3** by Spartan via ‘‘conformer distribution’’ function (conditions: starting with the above final structure; all constraints removed; MMFF energy minimization in vacuum) showing structural vibration of **3**. MC in red, tether in blue and C12 chains in grey.

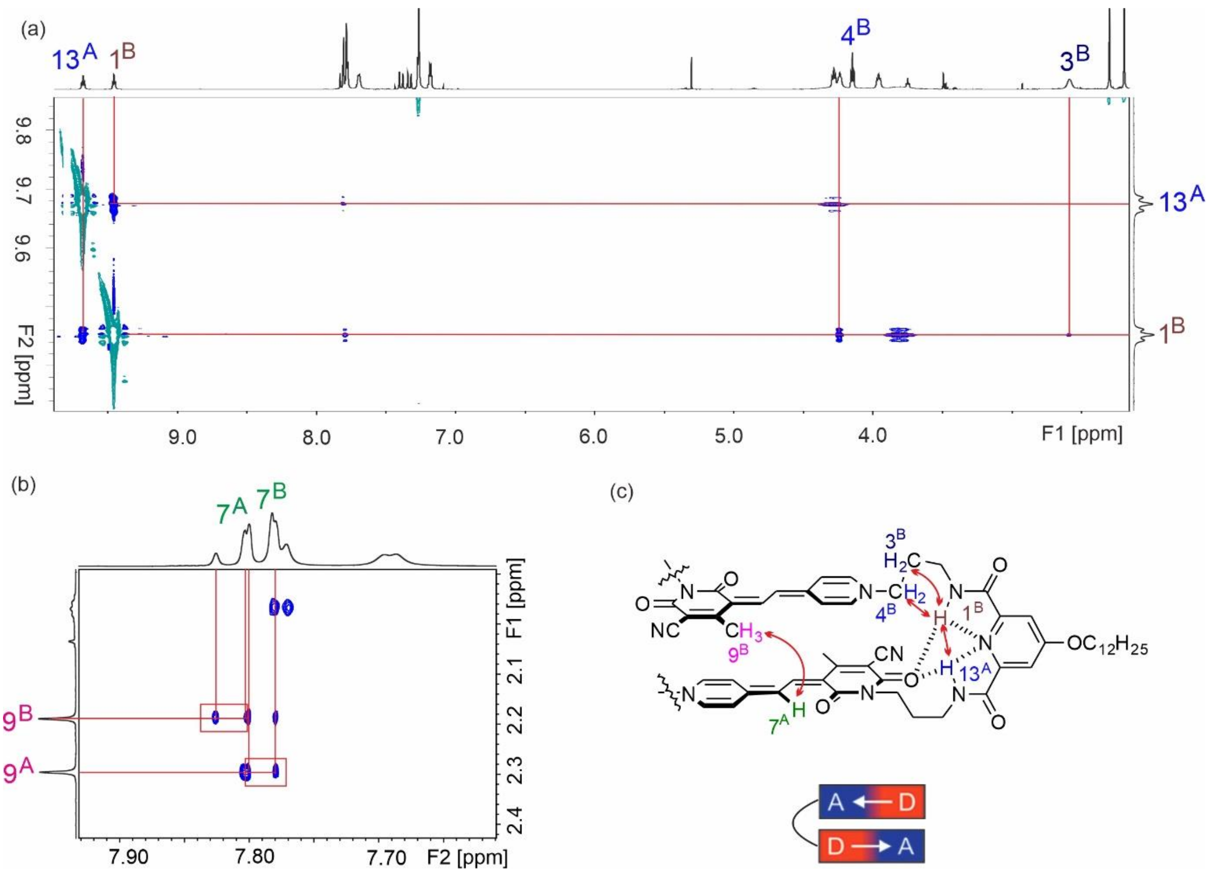

Fig. S7 (a, b) Excerpt of ^{1}H-^{1}H ROESY spectrum (600 MHz) of dimer **2** in $CDCl_3$ at 295 K. (c) Partial chemical structure of **2** with the correlations observed in the ^{1}H-^{1}H ROESY spectrum indicated by red double-headed arrows. Only one set of correlation is shown for clarity.

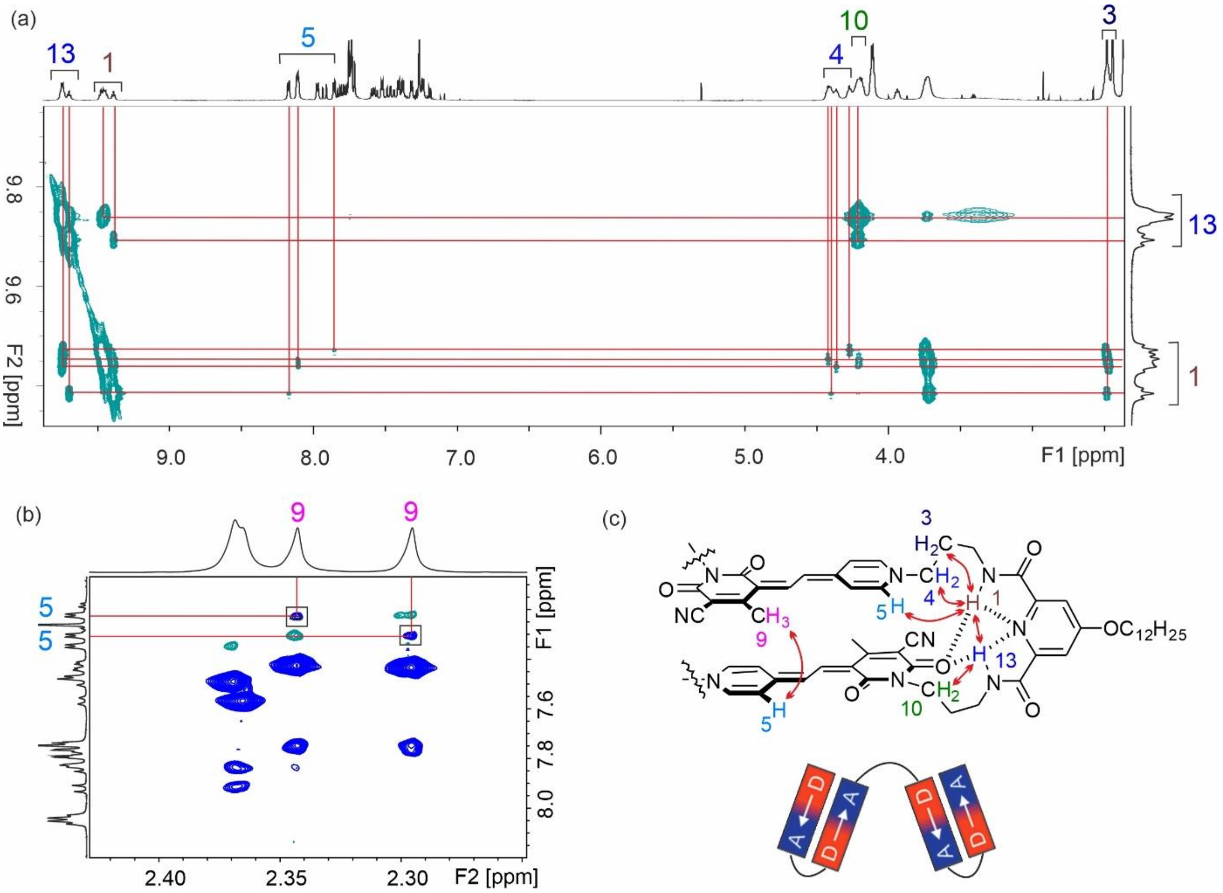

Fig. S8 (a, b) Excerpt of ^1H-^1H ROESY spectrum (600 MHz) of tetramer **4** in $CDCl_3$ at 295 K. (c) Partial chemical structure of **4** with the correlations observed in the ^1H-^1H ROESY spectrum indicated by red double-headed arrows. Only one set of correlation is shown for clarity.

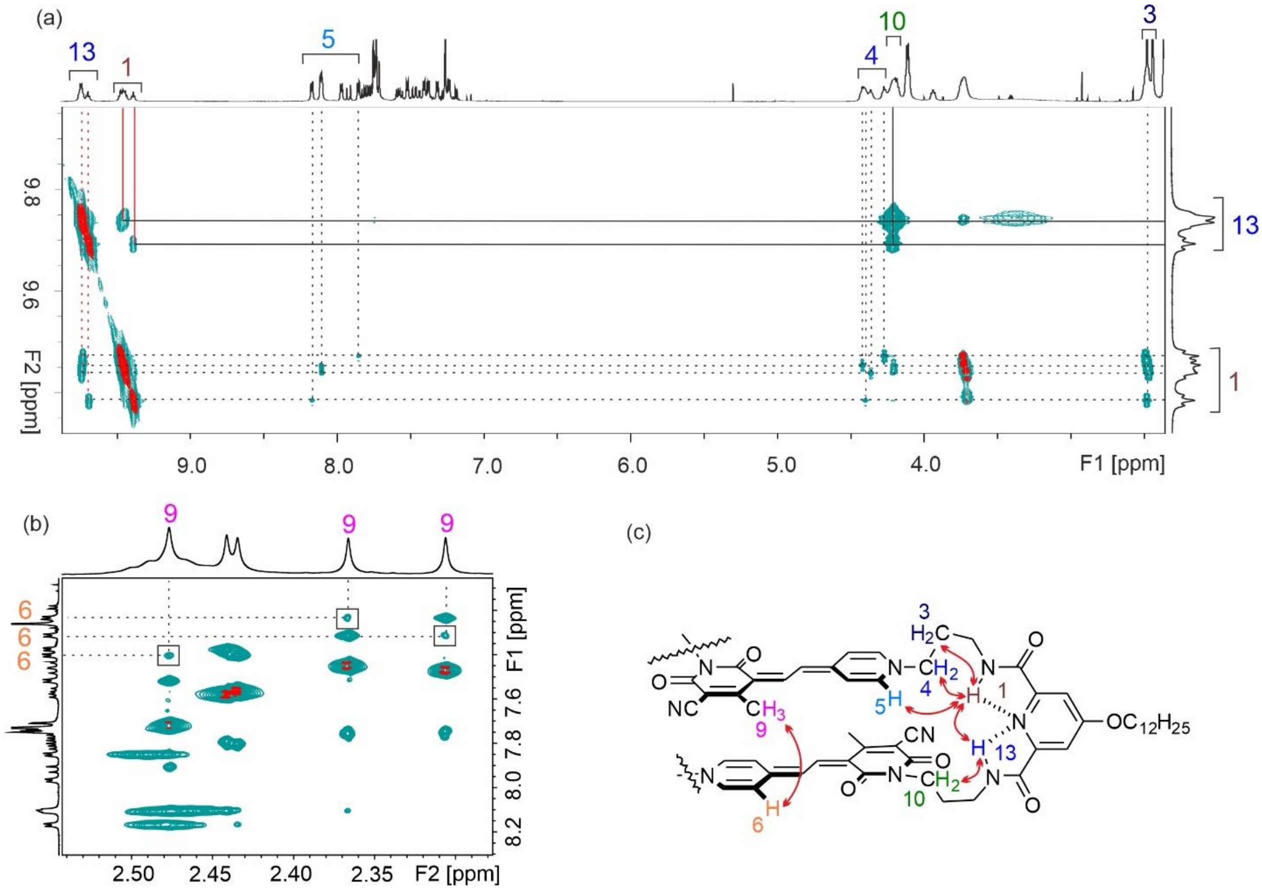

Fig. S9 (a, b) Excerpt of ^{1}H-^{1}H NOESY spectrum (600 MHz, green, mixing time = 300 ms) and ^{1}H-^{1}H-COSY (600 MHz, red) of pentamer **5** in $CDCl_3$ at 295 K. (c) Partial chemical structure of **5** with the correlations observed in the ^{1}H-^{1}H ROESY spectrum indicated by red double-headed arrows. Only one set of correlation is shown for clarity.

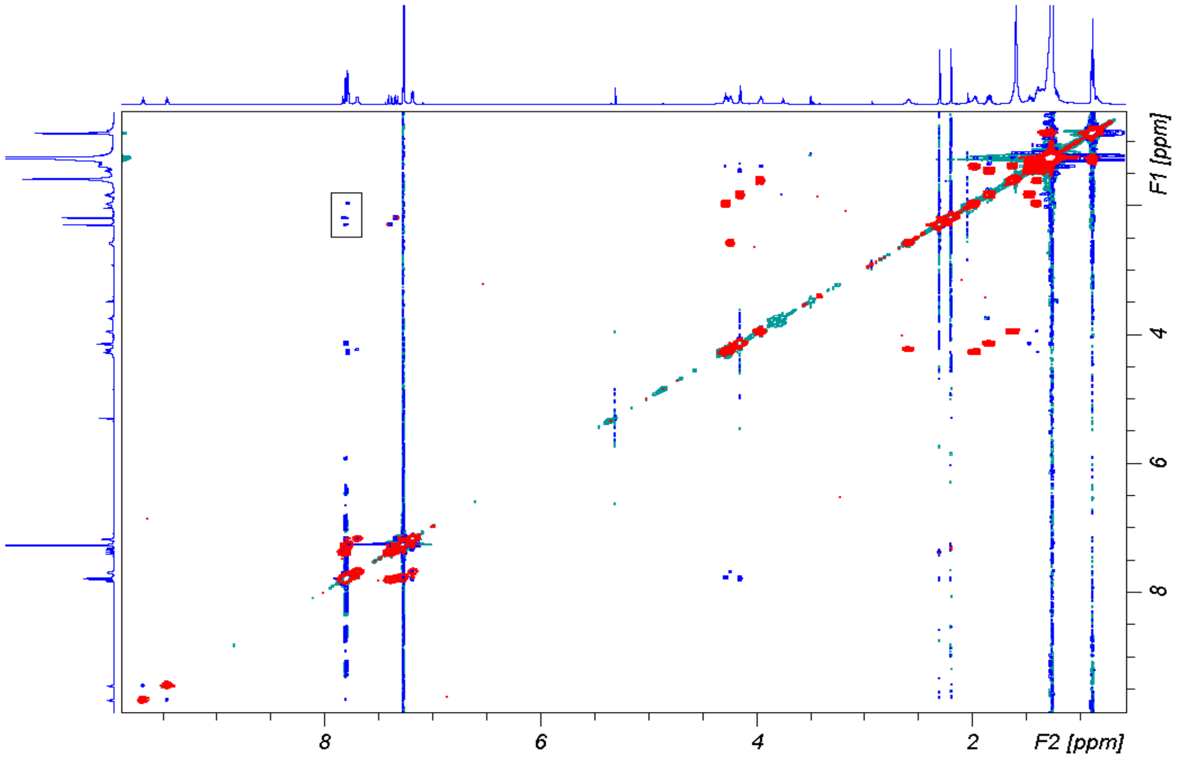

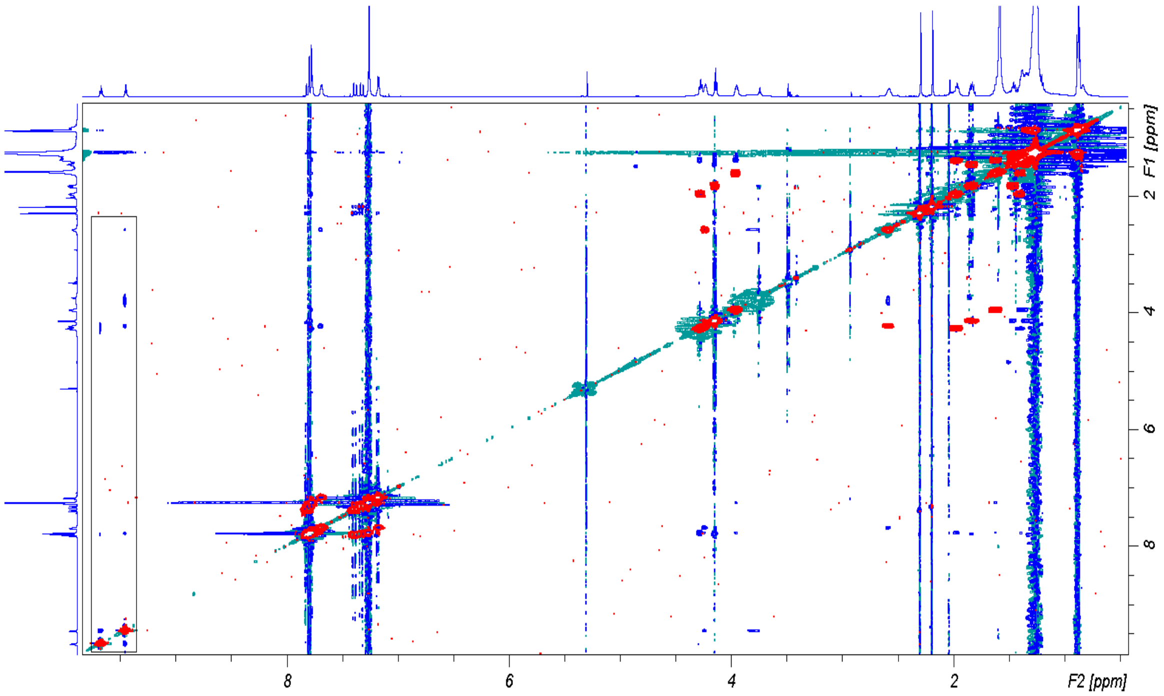

Fig. S10 Full ^{1}H-^{1}H ROESY (600 MHz, positive signals in blue and negative ones in green) and ^{1}H-^{1}H COSY (400 MHz, in red) spectra of dimer **2** in $CDCl_3$ at 295 K. The black rectangles include the investigated correlations. To properly show the investigated correlations, the upper graph is with lower signal intensity while the bottom one is with higher signal intensity.

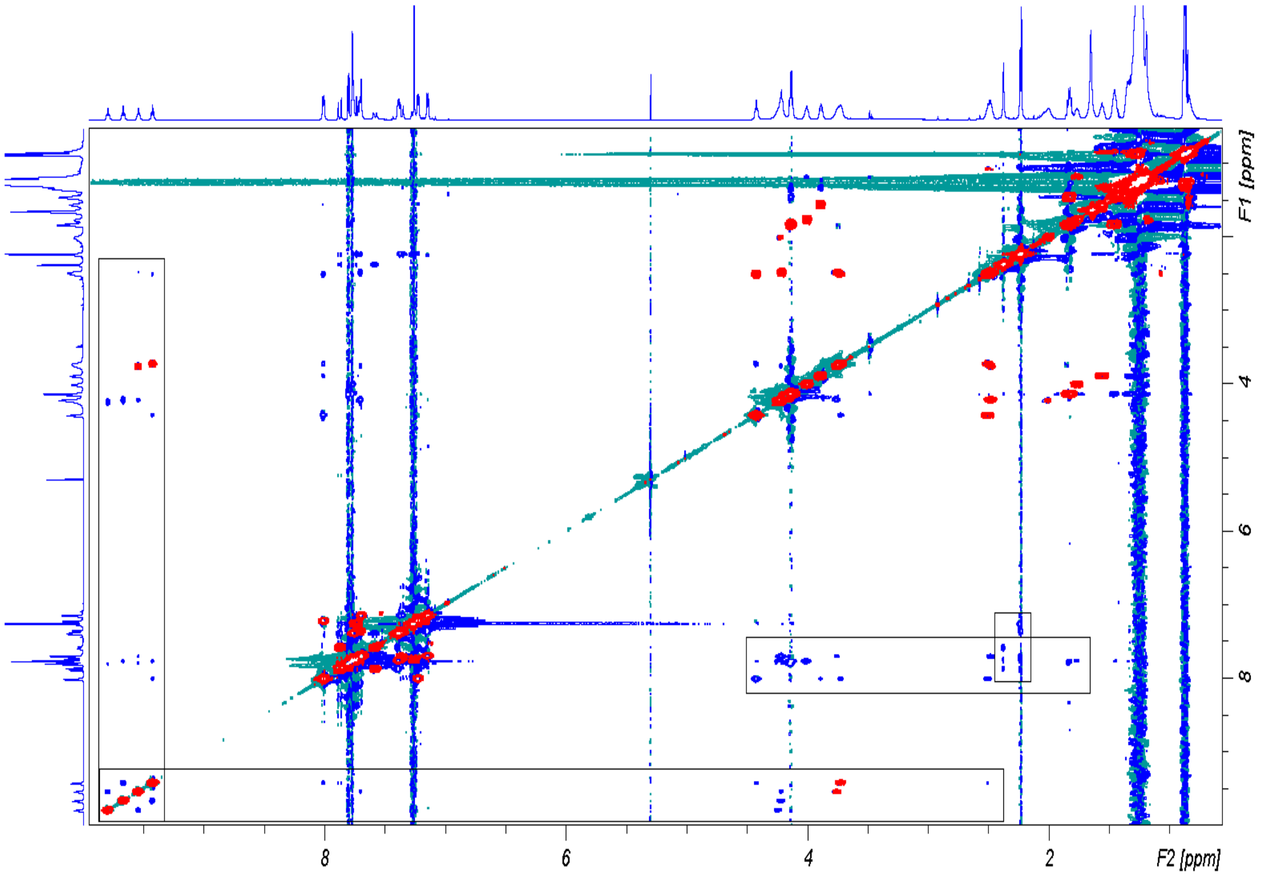

Fig. S11 Full ^{1}H-^{1}H ROESY (600 MHz, positive signals in blue and negative ones in green) and ^{1}H-^{1}H COSY (400 MHz, in red) spectra of trimer **3** in $CDCl_3$ at 295 K. The black rectangles include the investigated correlations.

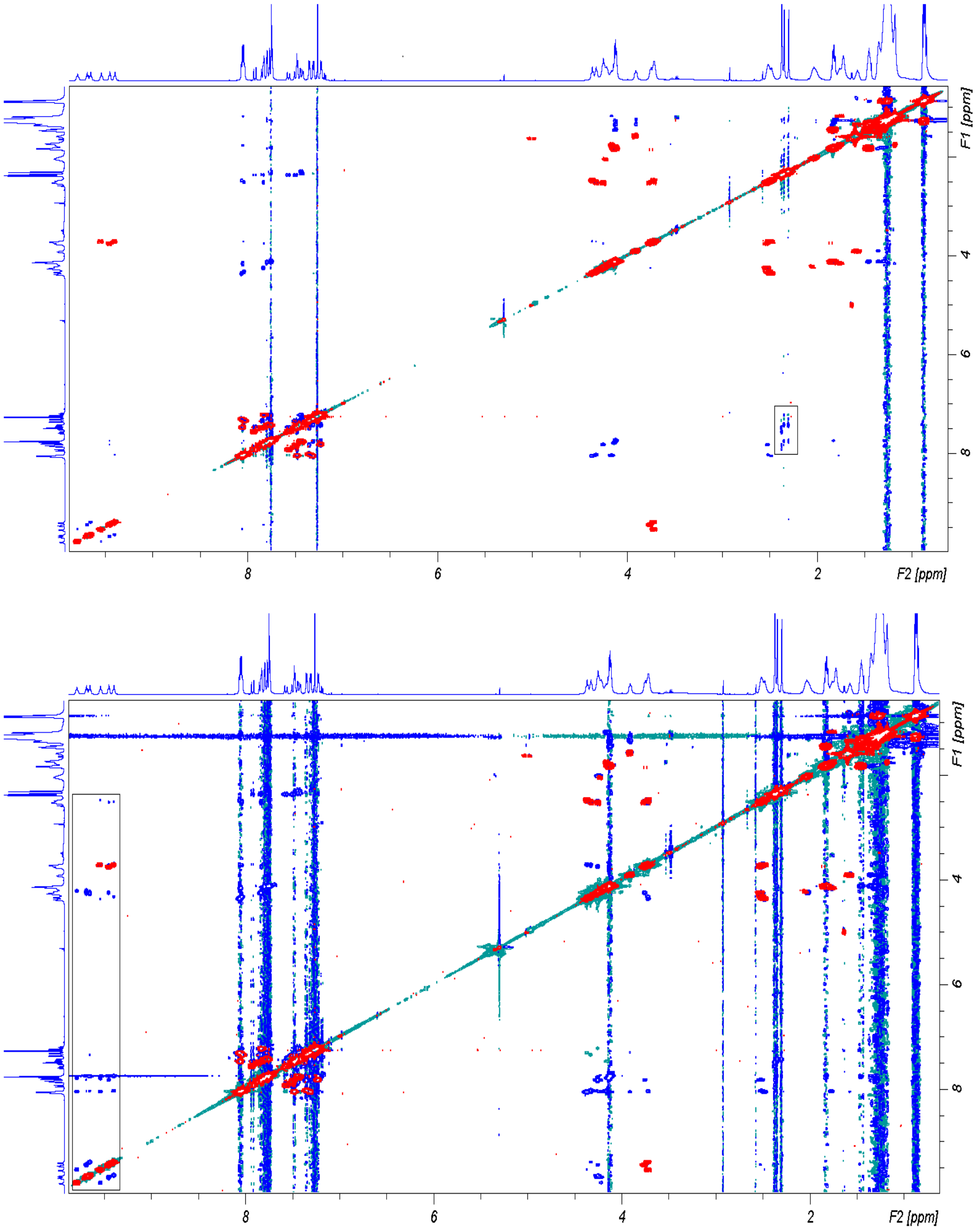

Fig. S12 Full ^{1}H-^{1}H ROESY (600 MHz, positive signals in blue and negative ones in green) and ^{1}H-^{1}H COSY (400 MHz, in red) spectra of tetramer **4** in $CDCl_3$ at 295 K. The black rectangles include the investigated correlations. To properly show the investigated correlations, the upper graph is with lower signal intensity while the bottom one is with higher signal intensity.

UV-vis and fluorescence studies

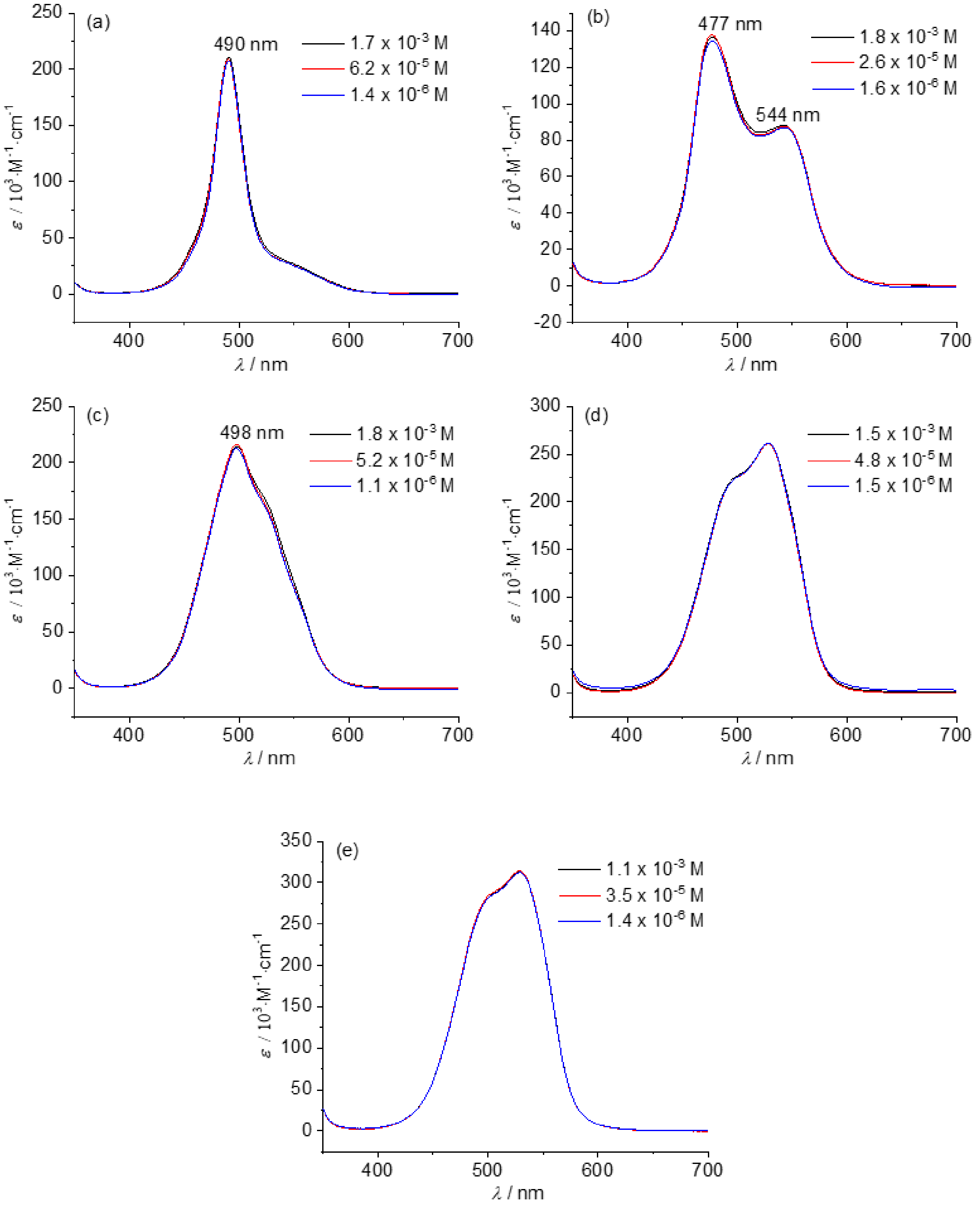

Fig. S13 Concentration-dependent UV-vis spectra of MC oligomers (a) **2**, (b) **3**, (c) **4**, (d) **5** and (e) **6** in $CHCl_3$ at 293 K.

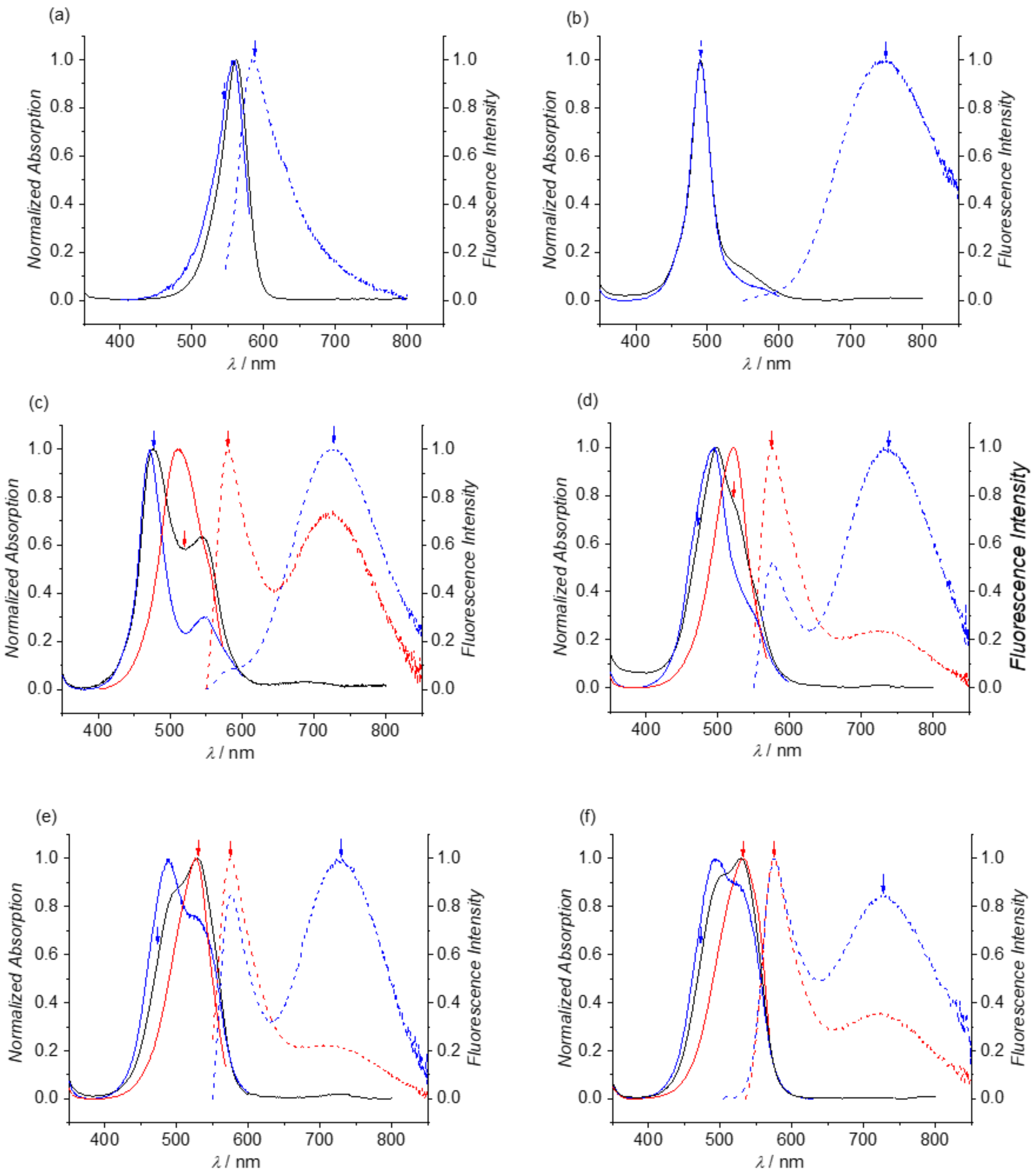

Fig. S14 Normalized UV/vis absorption (black), emission (red and blue dashed line), and excitation spectra (red and blue solid line) of (a) reference dye **1** at 3.5 x 10^{-6} M, (b) dimer **2** at 2.5 x 10^{-6} M, (c) trimer **3** at 3.0 x 10^{-6} M, (d) tetramer **4** at 2.5 x 10^{-6} M, (e) pentamer **5** at 2.0 x 10^{-6} M and hexamer **6** at 2.0 x 10^{-6} M in $CHCl_3$ at 293 K. Colored arrows indicate the excitation wavelength or the detection wavelength of the corresponding emission or excitation spectra, respectively, with the same colors.

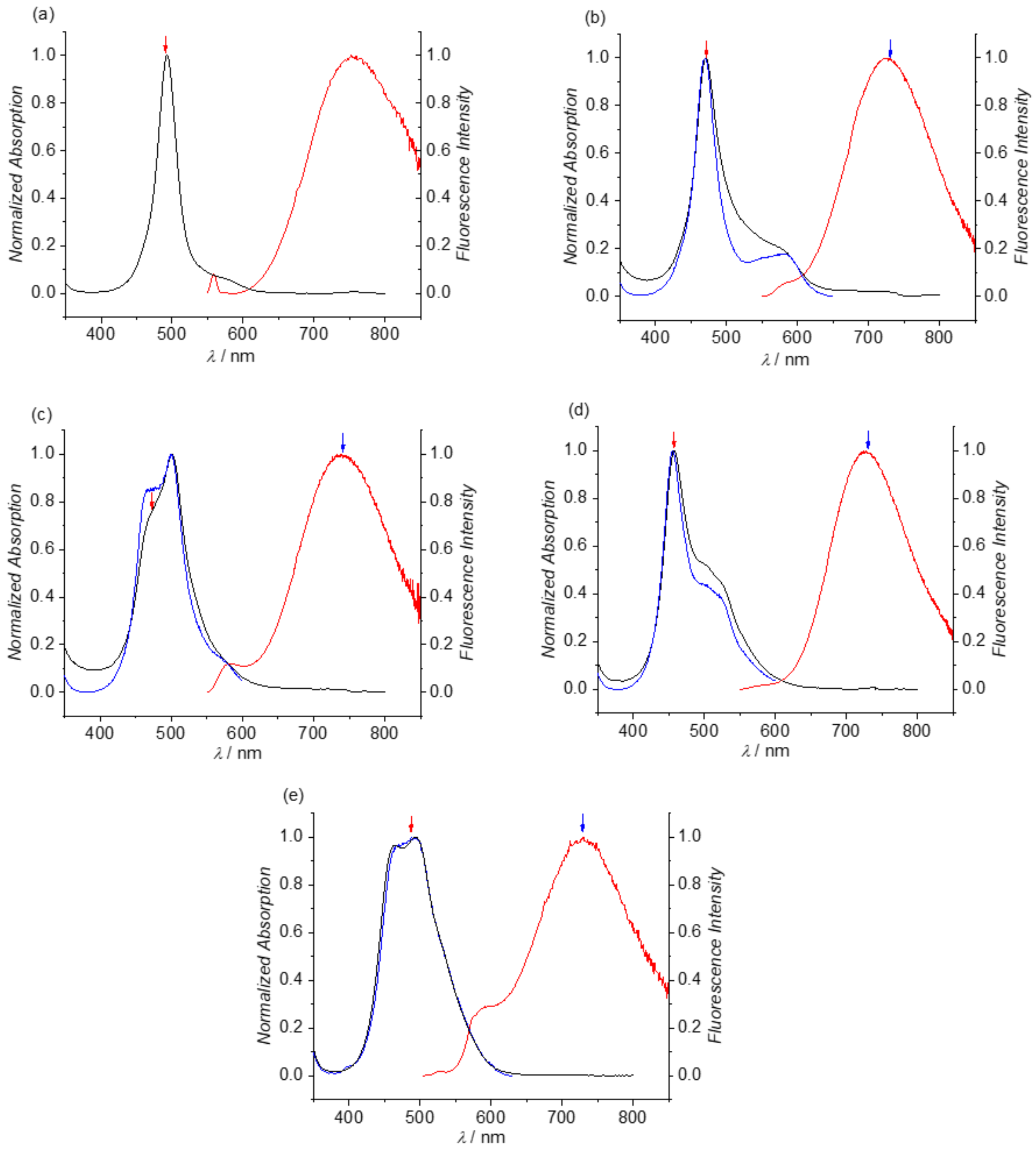

Fig. S15 Normalized UV/vis absorption (black), emission (red), and excitation spectra (blue) of (a) dimer **2** at 3.0 x 10^{-6} M, (b) trimer **3** at 2.5 x 10^{-6} M, (c) tetramer **4** at 2.5 x 10^{-6} M, (d) pentamer **5** at 3.0 x 10^{-7} M and (e) hexamer 6 at 4.0 x 10^{-7} M in $CHCl_3$/MCH 30:70 at 293 K. Colored arrows indicate the excitation wavelength or the detection wavelength of the corresponding emission or excitation spectra, respectively, with the same colors.

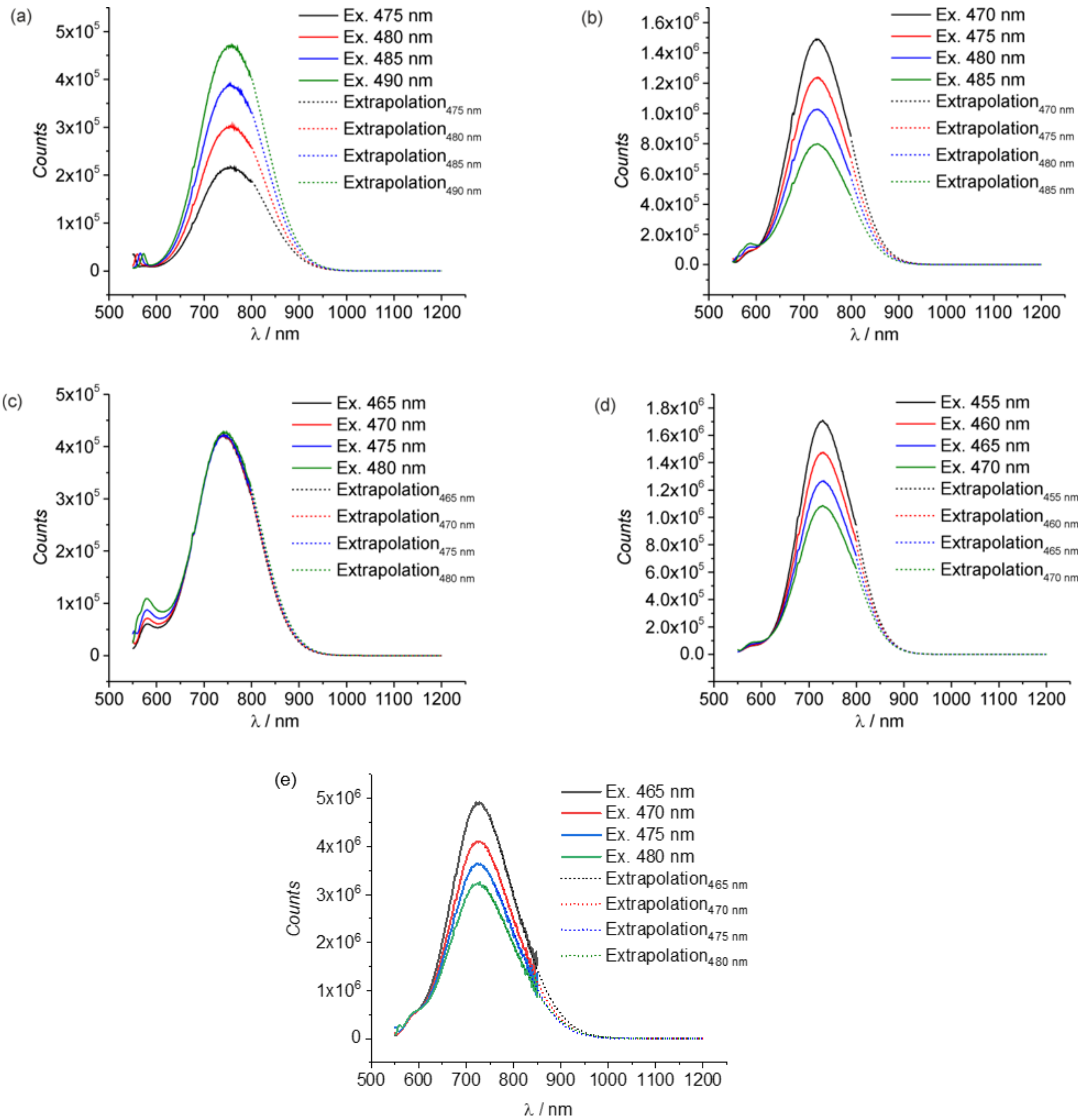

Fig. S16 Fluorescence spectra (solid lines) and extrapolation (Gaussian function, dotted lines) of (a) dimer **2** at 3.0×10^{-6} M, (b) trimer **3** at 2.5×10^{-6} M, (c) tetramer **4** at 2.5×10^{-7} M, (d) pentamer **5** at 3.0×10^{-6} M and (e) hexamer **6** at 2.5×10^{-6} M in $CHCl_3$/MCH 30:70 at 293 K.

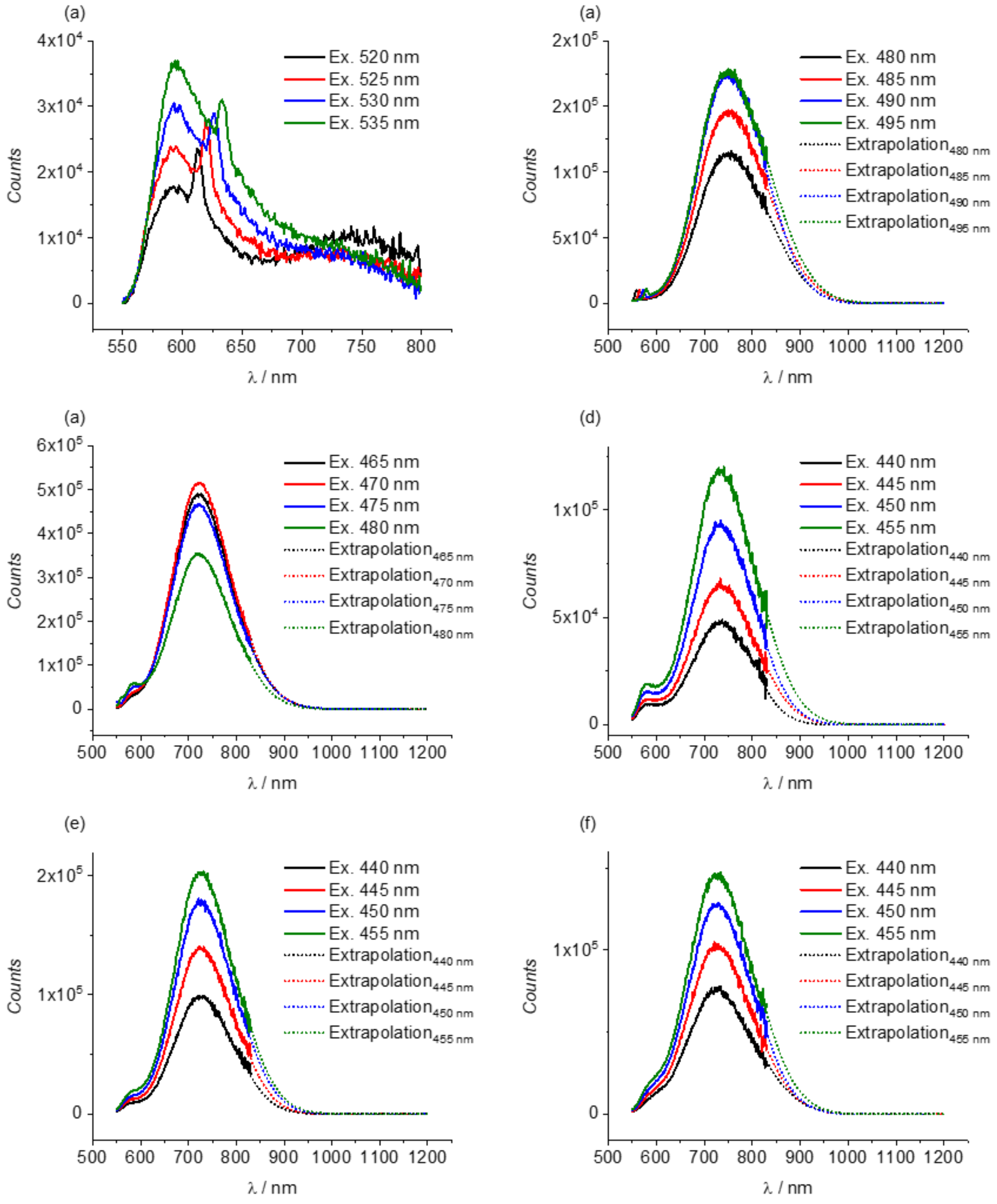

Fig. S17 Fluorescence spectra (solid lines) and extrapolation (Gaussian function, dotted lines) of (a) monomer **1**, (b) dimer **2**, (c) trimer **3**, (d) tetramer **4**, (e) pentamer **5** and (f) hexamer **6** in $CHCl_3$/liquid paraffin 30:70 at 293 K and optical densities ≤ 0.5.

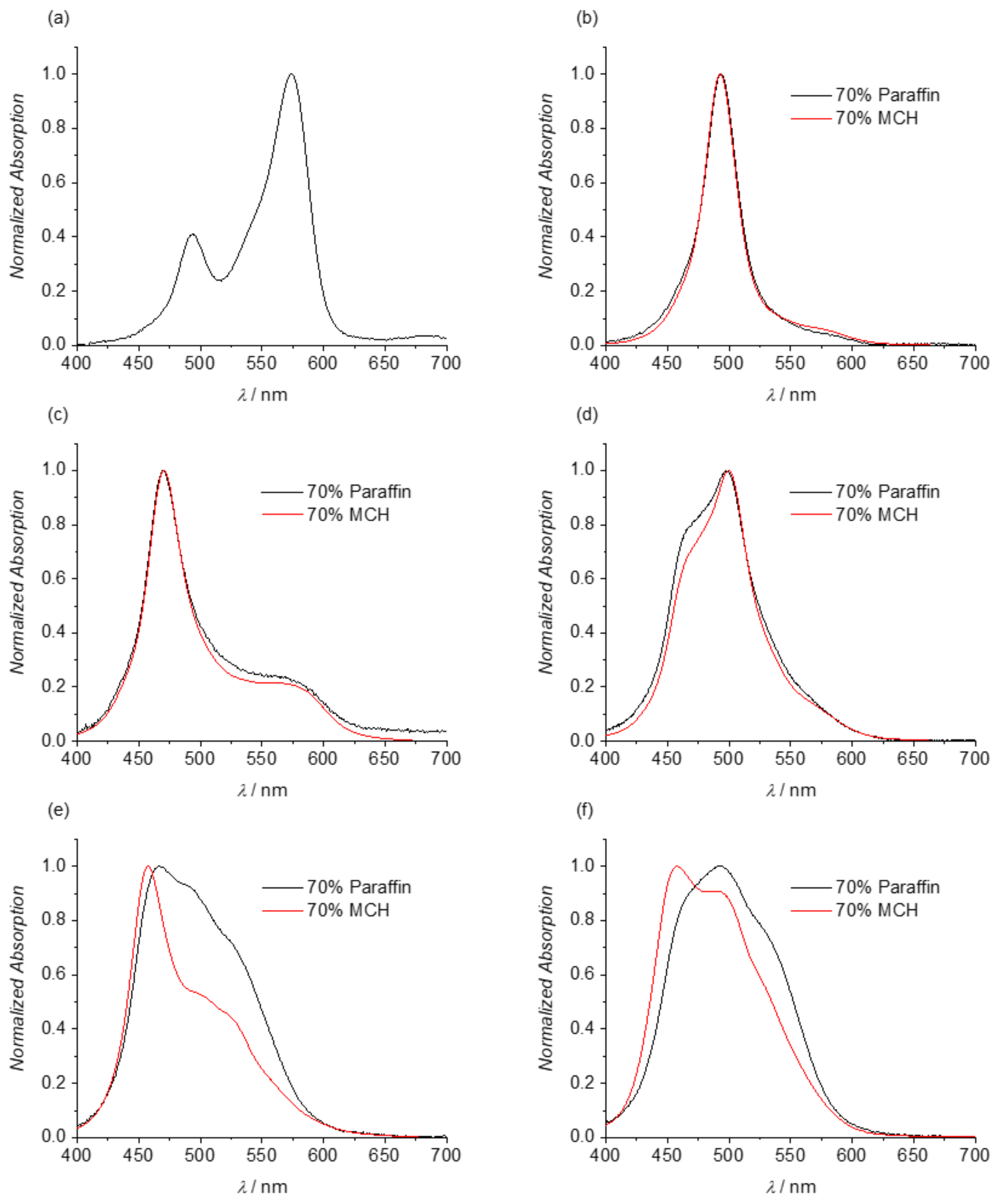

Fig. S18 Normalized absorption spectra of (a) monomer **1**, (b) dimer **2**, (c) trimer **3**, (d) tetramer **4**, (e) pentamer **5** and (f) hexamer **6** in $CHCl_3$/liquid paraffin 30:70 (black) and $CHCl_3$/MCH 30:70 (red) at 293 K.

Table S2 Fluorescence data of MC reference **1** and MC oligomers **2-6** in $CHCl_3$/MCH 30:70.

	Life time (τ) / ns	Average life time / ns	Quantum yield (Φ)[b]	k_r[c]/ $10^7 s^{-1}$	k_{nr}[c]/ $10^7 s^{-1}$
**1**	τ= 0.2[a] χ^2= 1.24	---	0.3% ± 0.07%[a]	1.5	500
**2**	τ_1= 2.9 (50%) τ_2= 5.6 (50%) χ^2= 1.03	τ_{avg} = 4.3	0.6% ± 0.1%	0.14	23
**3**	τ_1= 3.2 (51%) τ_2= 5.0 (49%) χ^2= 1.13	τ_{avg} = 4.1	2.1% ± 0.2%	0.51	24
**4**	τ_1= 2.9 (38%) τ_2= 5.3 (62%) χ^2= 1.07	τ_{avg} = 4.4	0.9% ± 0.1%	0.21	23
**5**	τ_1= 3.1 (40%) τ_2= 6.1 (60%) χ^2= 1.02	τ_{avg} = 4.9	1.7% ± 0.3%	0.35	20
**6**	τ_1= 1.9 (16%) τ_2= 5.1 (84%) χ^2= 1.07	τ_{avg} = 4.6	1.5% ± 0.2%	0.33	21

[a] Life time and quantum yield could not be determined for **1** in CHCl3/MCH (30:70) with our instrument due to the weak fluorescence. The values were determined in $CHCl_3$/liquid paraffin (30:70, Table S3).[b] Quantum yield was determined against *N*,*N*'-bis(2,6-diisopropylphenyl)-1,6,7,12-tetraphenoxy-3,4,9,10-perylenetetracarboxylic diimide in $CHCl_3$ as the reference and the emission spectra of **2-6** were extrapolated through Gaussian function before use. [c]Determined according to $k_r = \Phi/\tau$ and $k_{nr} = 1/\tau_{fl} - k_{fl}$.

Table S3 Fluorescence data of MC reference **1** and MC oligomers **2-6** in $CHCl_3$/liquid paraffin 30:70.

	Life time (τ) / ns	Average life time / ns	Quantum yield (Φ)[a]	k_r[b]/ $10^7 s^{-1}$	k_{nr}[b]/ $10^7 s^{-1}$
**1**	τ= 0.2 χ^2= 1.24	---	0.3% ± 0.07%	1.5	500
**2**	τ_1= 3.2 (54%) τ_2= 6.1 (46%) χ^2= 1.01	τ_{avg} = 4.5	1.1% ± 0.1%	0.24	22
**3**	τ_1= 3.6 (41%) τ_2= 5.8 (59%) χ^2= 1.04	τ_{avg} = 4.9	4.8% ± 0.1%	0.98	19
**4**	τ_1= 3.4 (42%) τ_2= 6.0 (58%) χ^2= 1.02	τ_{avg} = 4.9	1.9% ± 0.1%	0.39	20
**5**	τ_1= 2.6 (22%) τ_2= 5.9 (78%) χ^2= 1.01	τ_{avg} = 5.2	2.8% ± 0.1%	0.54	19
**6**	τ_1= 2.4 (23%) τ_2= 6.0 (77%) χ^2= 1.13	τ_{avg} = 5.2	2.1% ± 0.1%	0.40	19

[a]Quantum yield was determined against *N*,*N*'-bis(2,6-diisopropylphenyl)-1,6,7,12-tetraphenoxy-3,4,9,10-perylenetetracarboxylic diimide in $CHCl_3$ as the reference and the emission spectra of **2-6** were extrapolated through Gaussian function before use. [c]Determined according to $k_r = \Phi/\tau$ and $k_{nr} = 1/\tau_{fl} - k_{fl}$.

Calculations

Table S4 Oscillator strength *f* of the transitions and interfragment charge transfer (IFCT) analysis performed by the Multiwfn program[S12] between chromophore units 1 and 2 in the dimer **2** for the lowest 4 excited states.

	$S_0 \rightarrow S_1$	$S_0 \rightarrow S_2$	$S_0 \rightarrow S_3$	$S_0 \rightarrow S_4$
1→2	0.06	0.56	0.08	-0.73
f	0.0142	0.3075	2.1711	0.1202

Table S5 Oscillator strength *f* of the transitions and interfragment charge transfer (IFCT) analysis performed by the Multiwfn program[S12] between chromophore units 1, 2 and 3 in the trimer **3** for the lowest 7 excited states.

	$S_0 \rightarrow S_1$	$S_0 \rightarrow S_2$	$S_0 \rightarrow S_3$	$S_0 \rightarrow S_4$	$S_0 \rightarrow S_5$	$S_0 \rightarrow S_6$	$S_0 \rightarrow S_7$
1→2	0.00	0.05	-0.02	0.31	0.05	-0.02	-0.38
1→3	0.00	0.05	-0.32	-0.11	0.61	-0.03	0.01
2→3	0.00	0.02	-0.14	-0.19	0.08	-0.01	0.12
f	0.2596	0.0153	1.1895	0.1693	0.0299	1.1563	0.9892

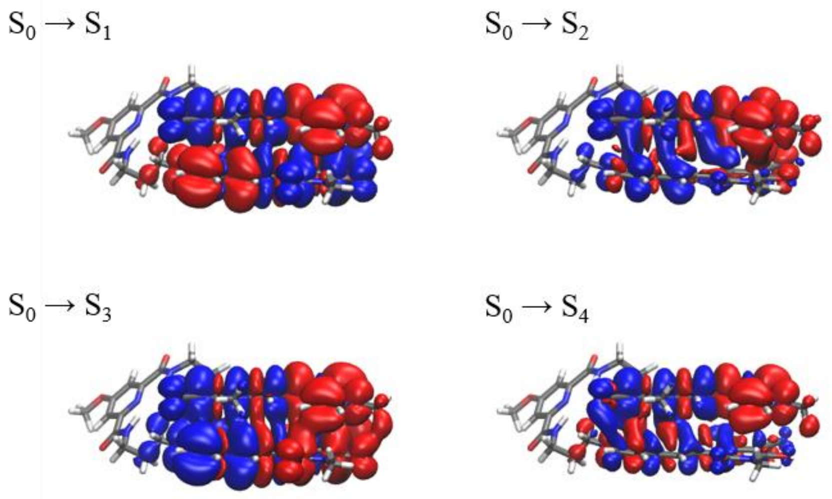

Fig. S19 Plotted TD-DFT transition densities of **2**.

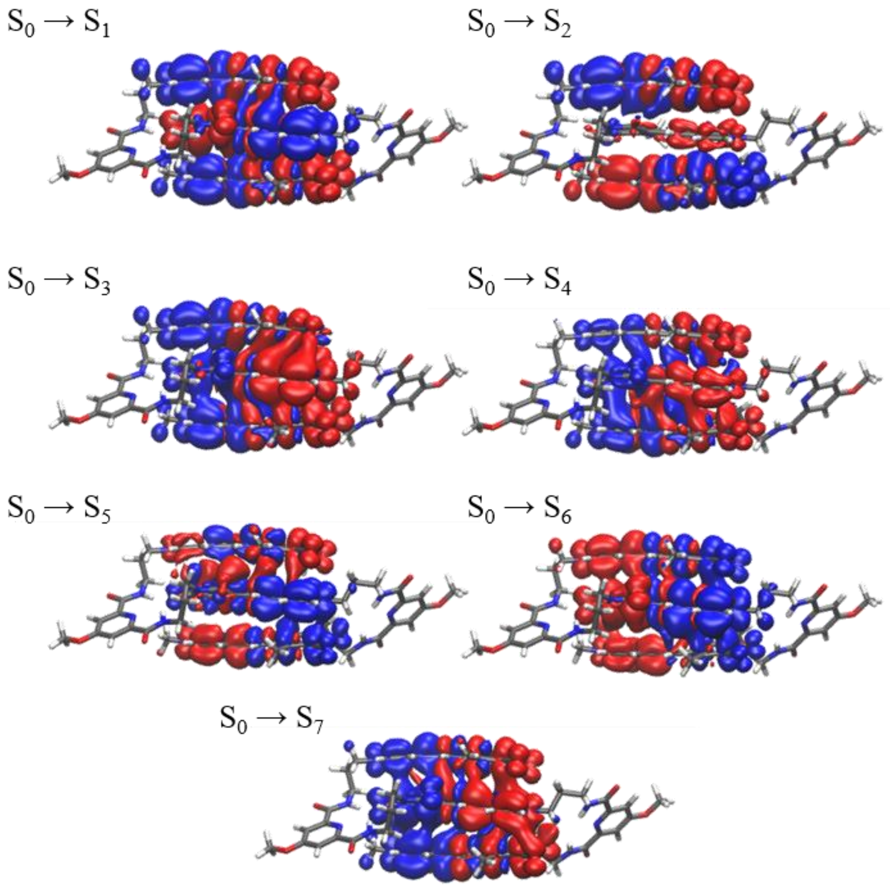

Fig. S20 Plotted TD-DFT transition densities of **3**.

1D NMR spectra

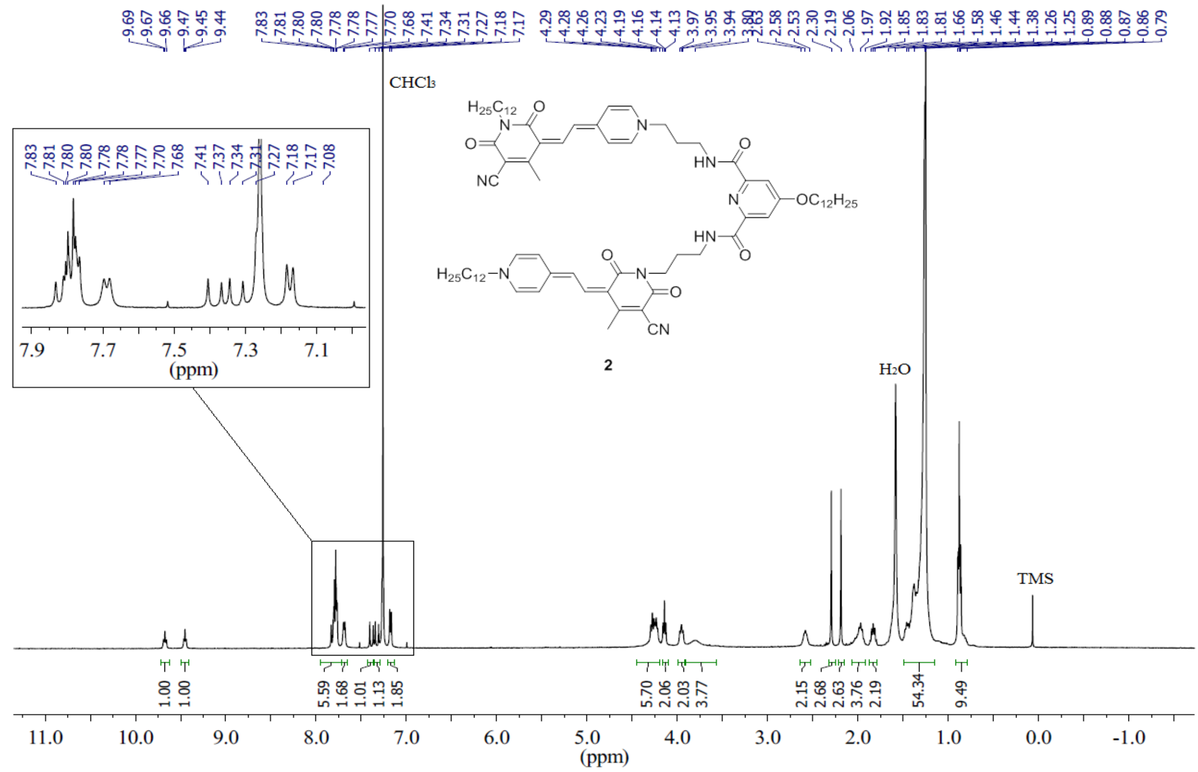

Fig. S21 ^{1}H NMR (400 MHz, $CDCl_3$) spectrum of the dimer **2** at 295 K.

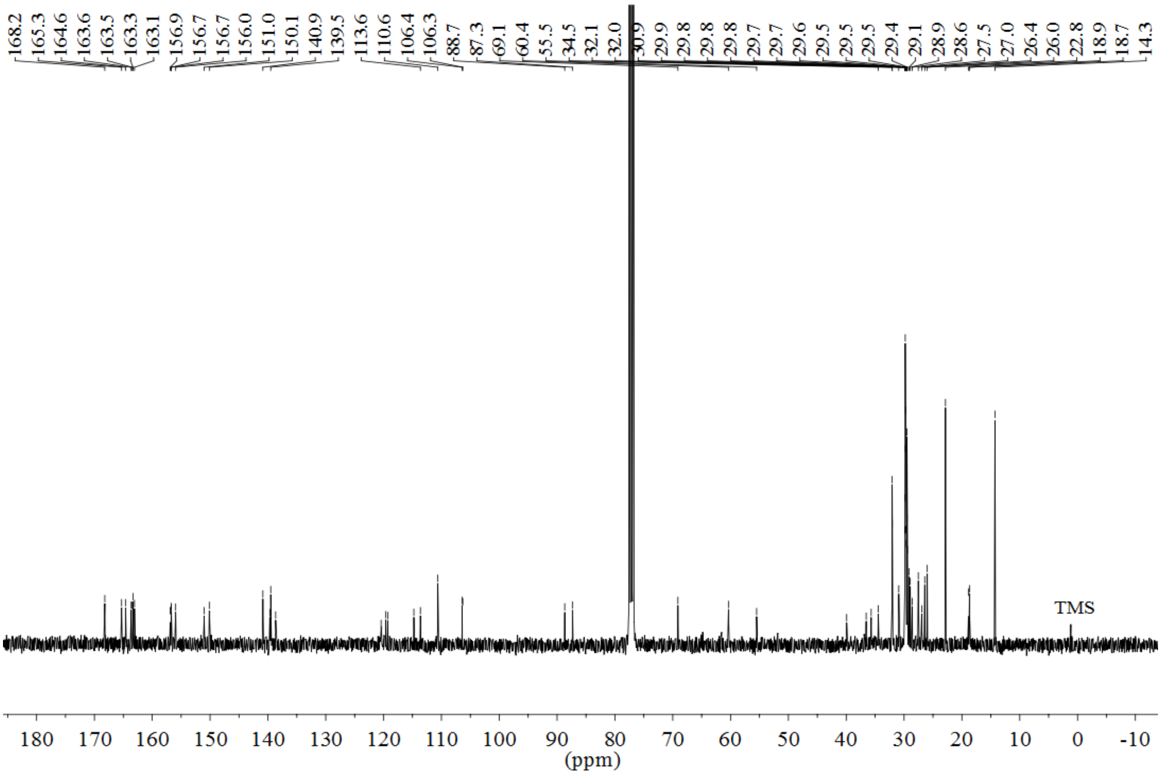

Fig. S22 ^{13}C NMR (101 MHz, $CDCl_3$) spectrum of the dimer **2** at 295 K.

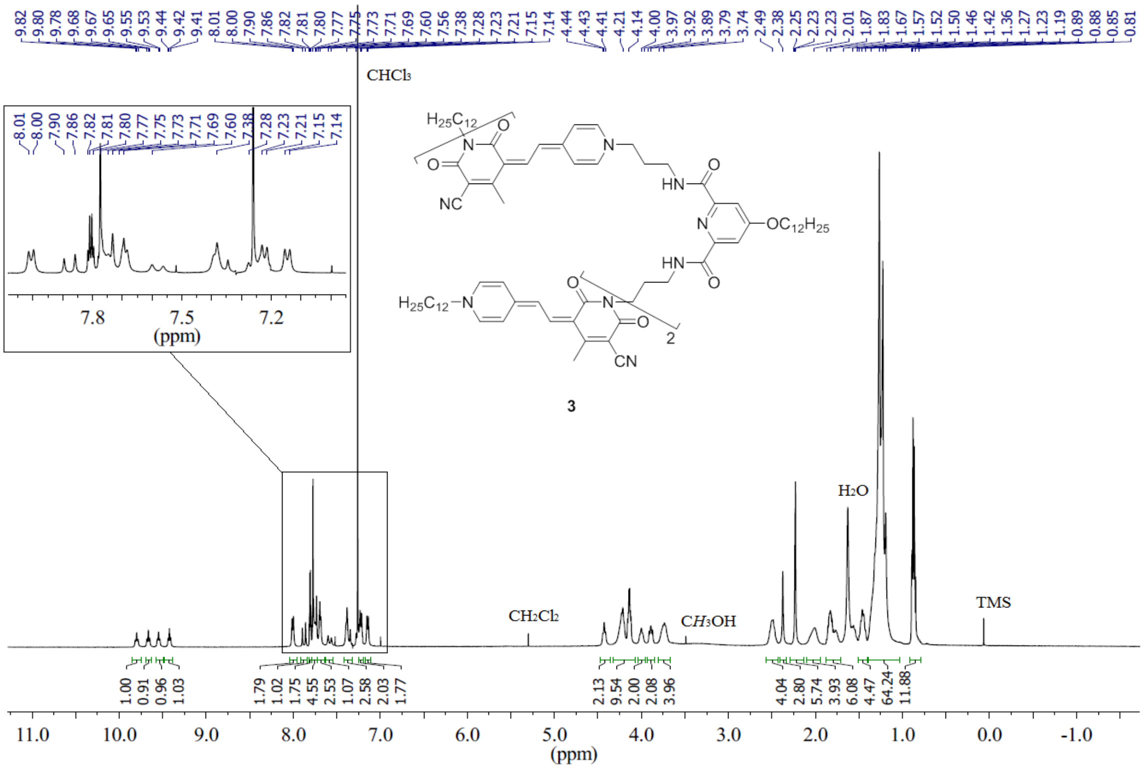

Fig. S23 ^{1}H NMR (400 MHz, $CDCl_3$) spectrum of the trimer **3** at 295 K.

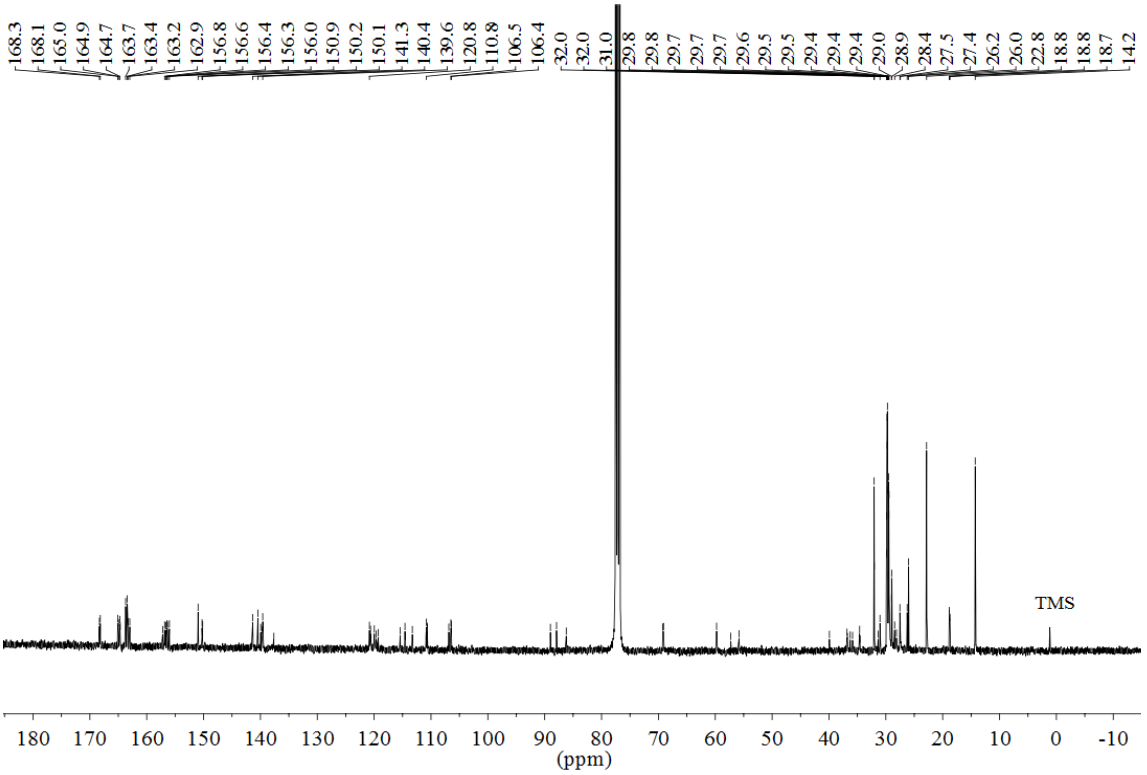

Fig. S24 ^{13}C NMR (101 MHz, $CDCl_3$) spectrum of the trimer **3** at 295 K.

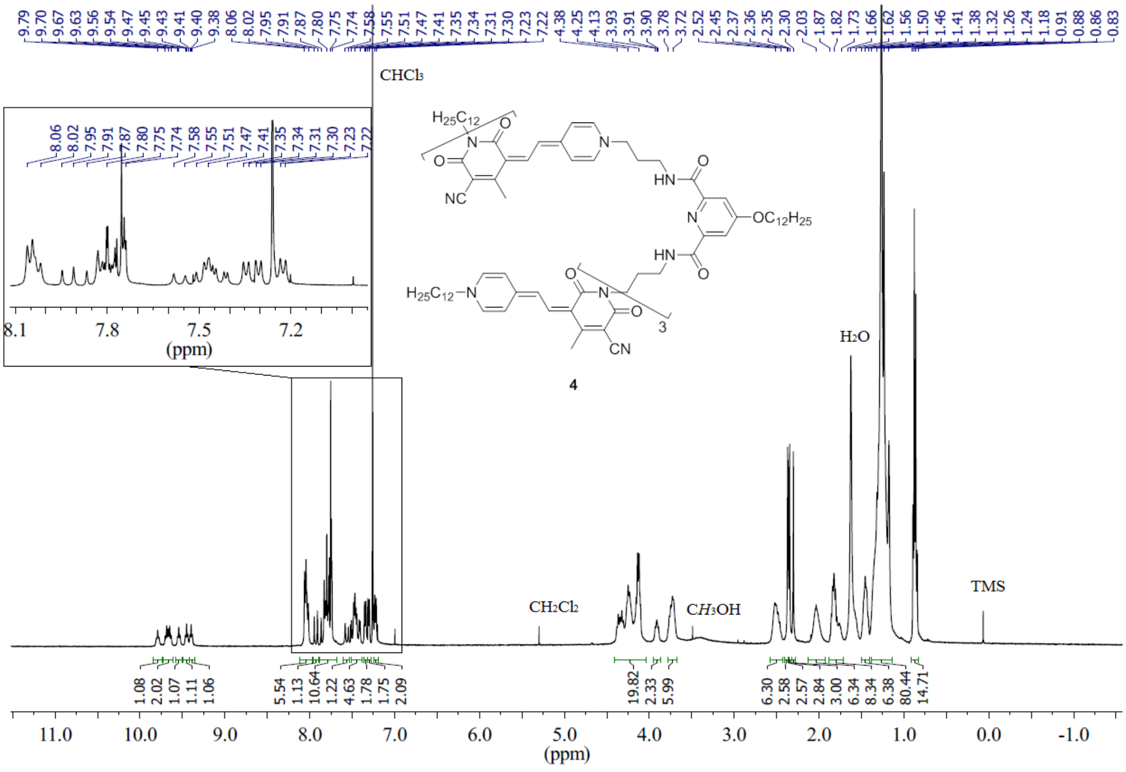

Fig. S25 ^{1}H NMR (400 MHz, $CDCl_3$) spectrum of the tetramer **4** at 295 K.

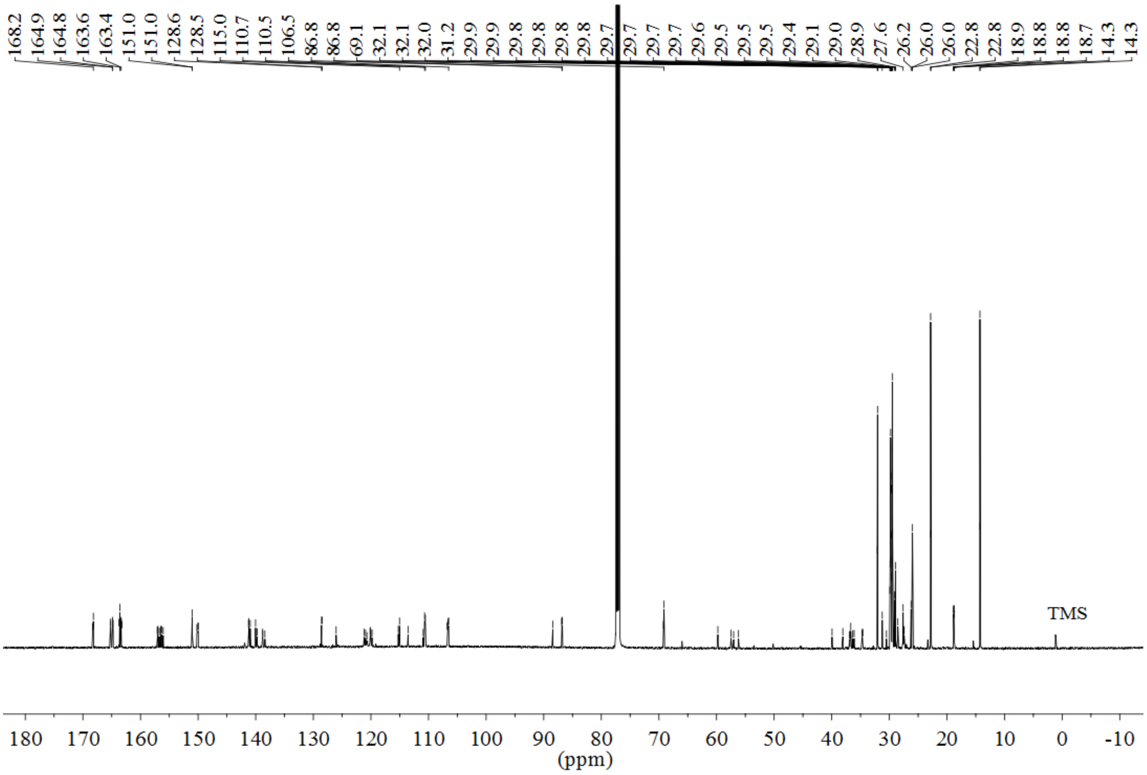

Fig. S26 ^{13}C NMR (101 MHz, $CDCl_3$) spectrum of the tetramer **4** at 295 K.

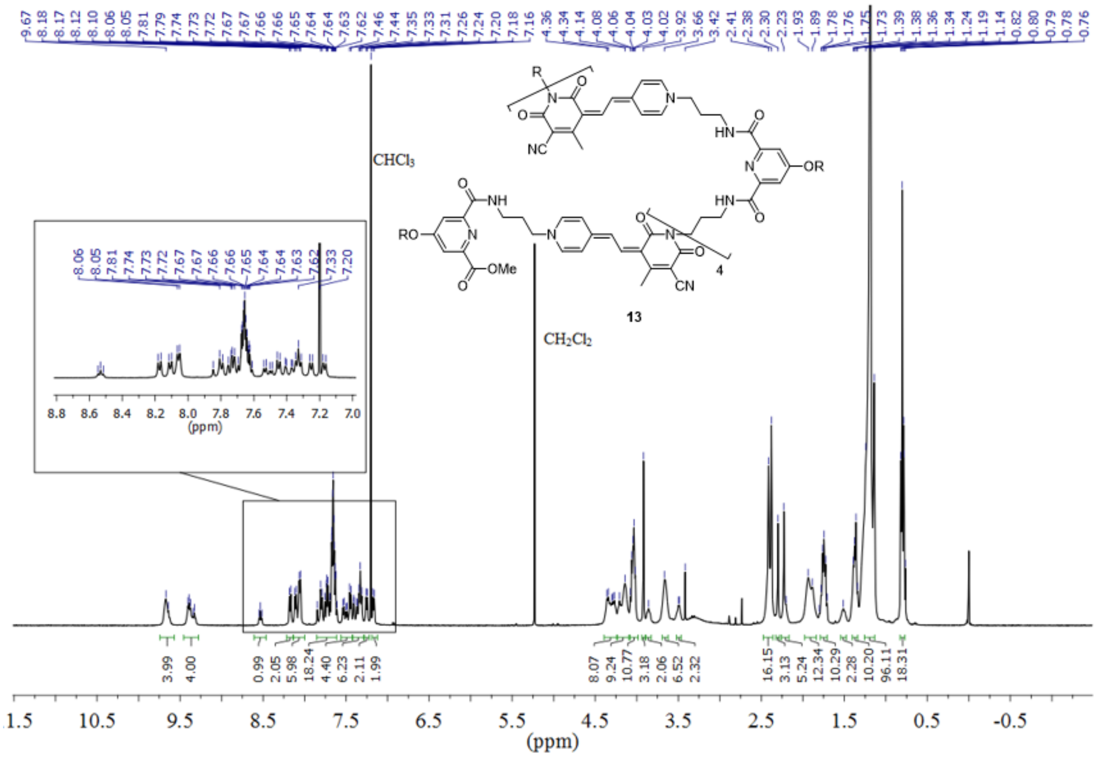

Fig. S27 ^{1}H NMR (400 MHz, $CDCl_3$) spectrum of **13** at 295 K.

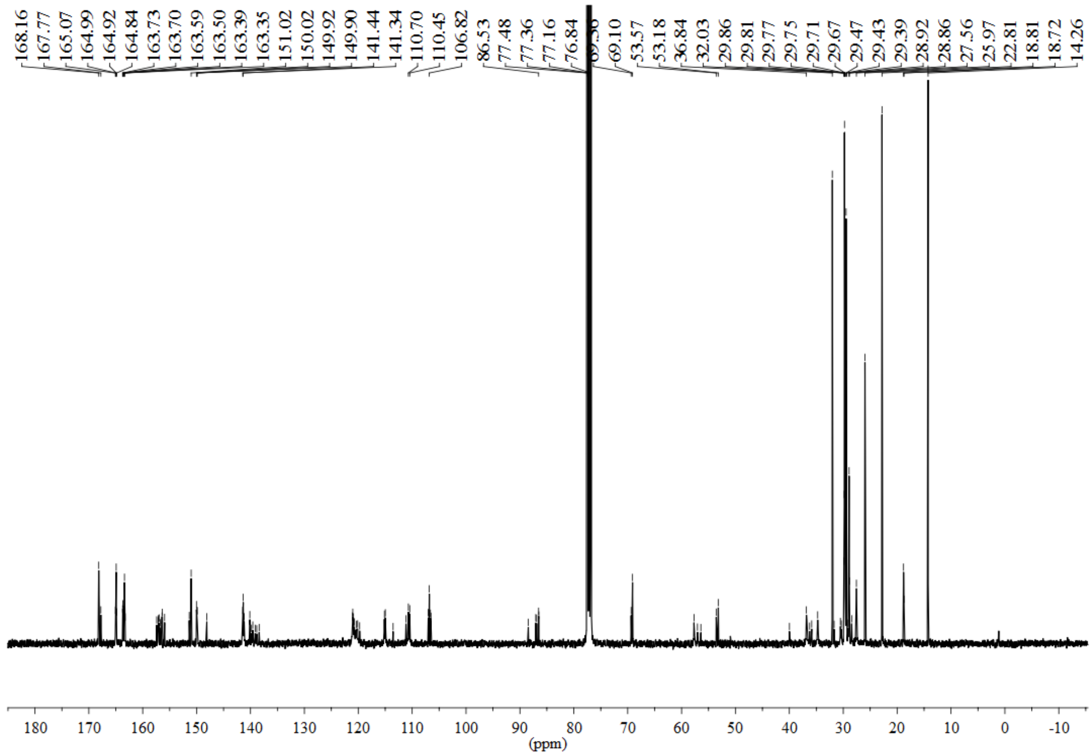

Fig. S28 ^{13}C NMR (101 MHz, $CDCl_3$) spectrum of **13** at 295 K.

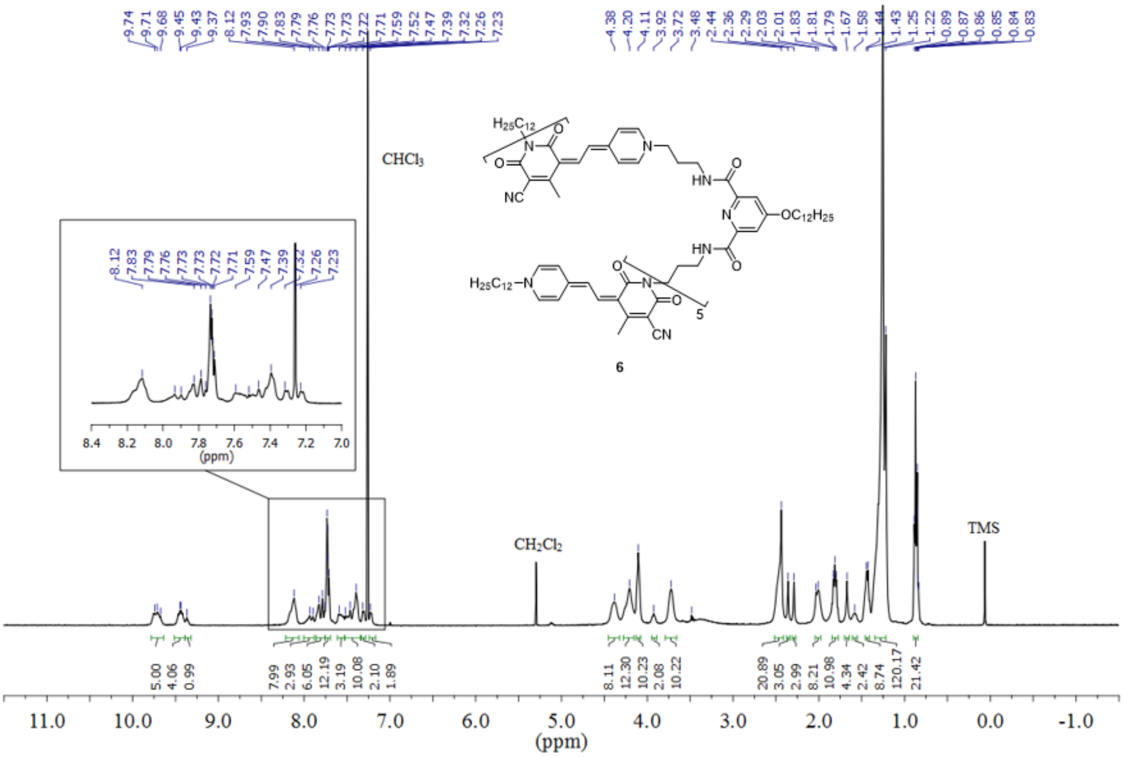

Fig. S29 ^{1}H NMR (400 MHz, $CDCl_3$) spectrum of the hexamer **6** at 295 K.

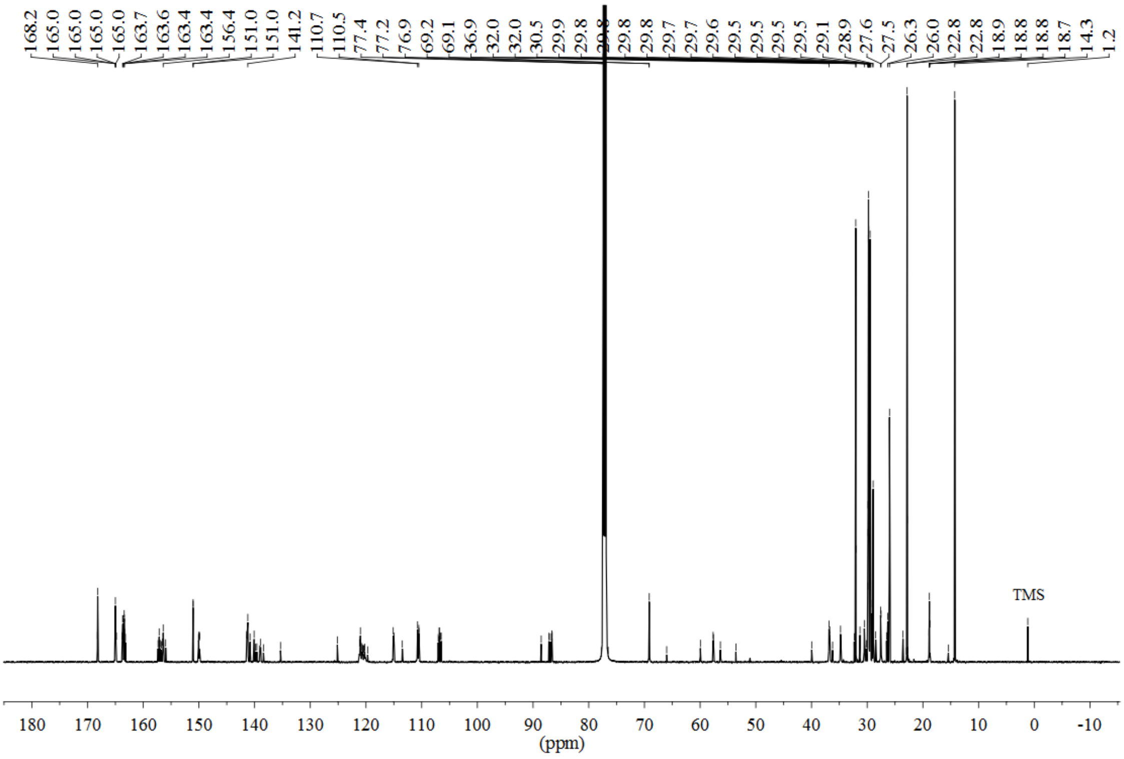

Fig. S30 ^{13}C NMR (101 MHz, $CDCl_3$) spectrum of the tetramer **4** at 295 K.

Mass spectra

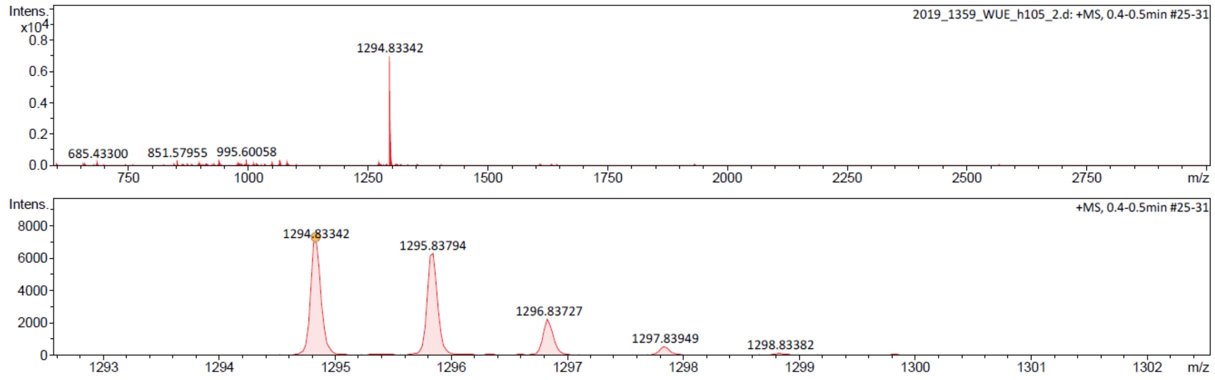

Meas. m/z	#	Ion Formula	m/z	err [ppm]	mSigma	# mSigma	Score	rdb	e¯ Conf	N-Rule
1294.83342	1	C77H109N9NaO7	1294.83422	0.62	44.0	1	100.00	27.5	even	ok

Fig. S31 HRMS (ESI-TOF, MeCN/$CHCl_3$) of **2** $[M + Na]^+$.

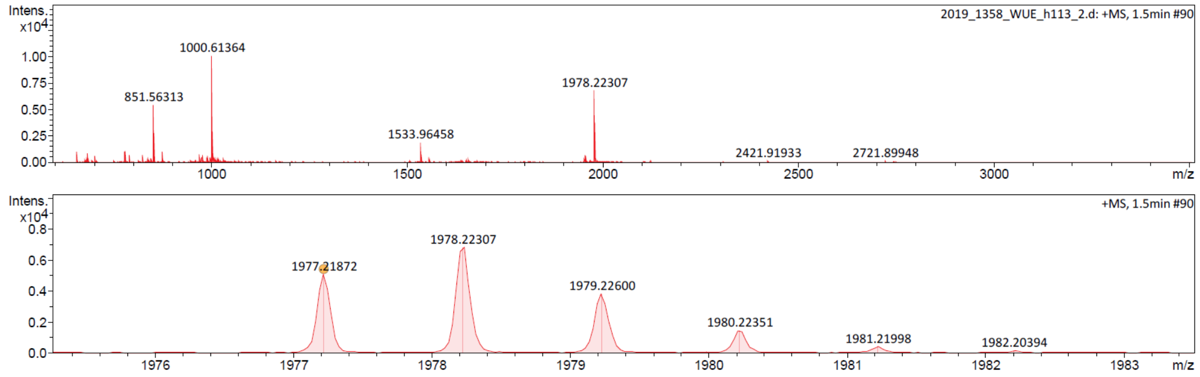

Meas. m/z	#	Ion Formula	m/z	err [ppm]	mSigma	# mSigma	Score	rdb	e¯ Conf	N-Rule
1977.21872	1	C116H159N15NaO12	1977.21849	-0.12	67.3	1	100.00	44.5	even	ok

Fig. S32 HRMS (ESI-TOF, MeCN/$CHCl_3$) of **3** $[M + Na]^+$.

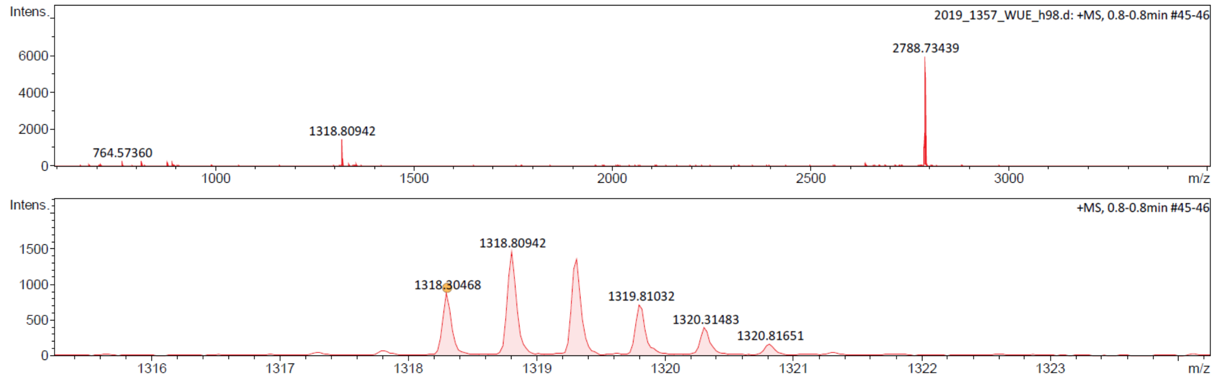

Meas. m/z	#	Ion Formula	m/z	err [ppm]	mSigma	# mSigma	Score	rdb	e¯ Conf	N-Rule
1318.30468	1	C155H209N21O17	1318.30622	1.17	33.4	1	100.00	62.0	even	ok

Fig. S33 HRMS (ESI-TOF, MeCN/$CHCl_3$) of **4** $[M]^{2+}$.

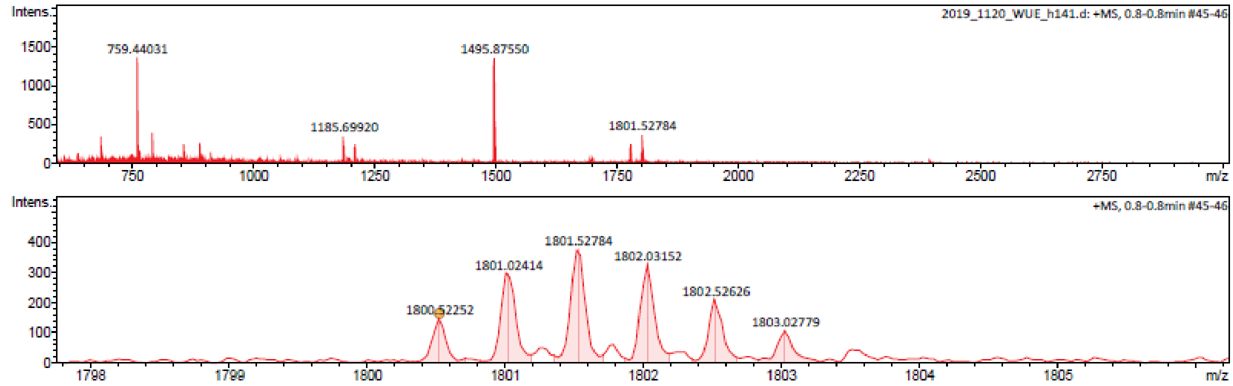

Meas. m/z	#	Ion Formula	m/z	err [ppm]	mSigma	# mSigma	Score	rdb	e¯ Conf	N-Rule
1800.52252	1	C205H271N29Na2O26	1800.52798	3.03	55.8	1	100.00	84.0	even	ok

Fig. S34 HRMS (ESI-TOF, MeCN/$CHCl_3$) of **13** $[M+2Na]^{2+}$.

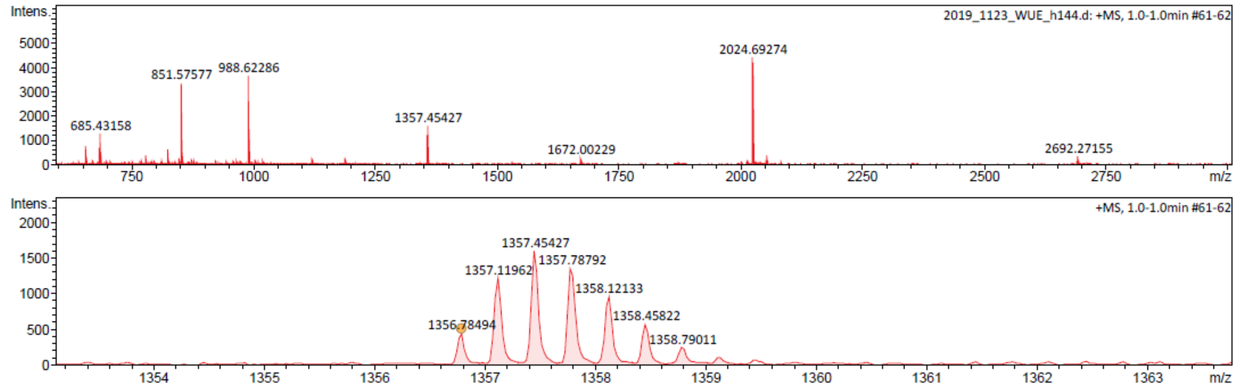

Meas. m/z	#	Ion Formula	m/z	err [ppm]	mSigma	# mSigma	Score	rdb	e¯ Conf	N-Rule
1356.78494	1	C233H309N33Na3O27	1356.78324	-1.25	29.2	1	100.00	94.5	even	ok

Fig. S35 HRMS (ESI-TOF, MeCN/$CHCl_3$) of **6** $[M+3Na]^{3+}$.

References

S1 G. Seybold and G. Wagenblast, *Dyes Pigments*, 1989, **11**, 303–317.

S2 T. L. Hwang and A. J. Shaka, *J. Am. Chem. Soc.*, 1992, **114**, 3157–3159.

S3 T.-L. Hwang, M. Kadkhodaei, A. Mohebbi and A. J. Shaka, *Magn. Reson. Chem.*, 1992, **30**, S24–S34.

S4 E. Ämmälahti, M. Bardet, D. Molko and J. Cadet, *J. Magn. Reson. Series A* 1996, **122**, 230–232.

S5 A. D. Becke, *J. Chem. Phys.*, 1993, **98**, 1372-1377; C. Lee, W. Yang and R. G. Parr, *Physical Review B*, 1988, **37**, 785–789.

S6 F. Weigend and R. Ahlrichs, *Phys. Chem. Chem. Phys.*, 2005, **7**, 3297–3305.

S7 TURBOMOLE V6.5 2013, a development of University of Karlsruhe and Forschungszentrum Karlsruhe GmbH, 1989-2007, TURBOMOLE GmbH, since 2007; available from http://www.turbomole.com.

S8 Gaussian 16, Revision A.03, M. J. Frisch, et al., Gaussian, Inc., Wallingford CT, 2016.

S9 T. Yanai, D. P. Tew and N. C. Handy, *Chem. Phys. Lett.*, 2004, **393**, 51–57.

S10 S. Grimme, J. Antony, S. Ehrlich and H. Krieg, *J. Chem. Phys.* , 2010, **132**, 154104.

S11 X. Hu, J. O. Lindner and F. Würthner, *J. Am. Chem. Soc.*, 2020, **142**, 3321–3325.

S12 T. Lu and F. Chen, *J. Comput. Chem.*, 2012, **33**, 580–592.
